# Motivation for aggressive religious radicalization: goal regulation theory and a personality × threat × affordance hypothesis

**DOI:** 10.3389/fpsyg.2015.01325

**Published:** 2015-09-15

**Authors:** Ian McGregor, Joseph Hayes, Mike Prentice

**Affiliations:** ^1^Department of Psychology, University of Waterloo, Waterloo, ONCanada; ^2^Department of Psychology, University of SalzburgSalzburg, Austria

**Keywords:** religion, radicalization, aggression, approach motivation, avoidance motivation, anxiety, groups

## Abstract

A new set of hypotheses is presented regarding the cause of aggressive religious radicalization (ARR). It is grounded in classic and contemporary theory of human motivation and goal regulation, together with recent empirical advances in personality, social, and neurophysiological psychology. We specify personality traits, threats, and group affordances that combine to divert normal motivational processes toward ARR. Conducive personality traits are oppositional, anxiety-prone, and identity-weak (i.e., morally bewildered). Conducive threats are those that arise from seemingly insurmountable external forces and frustrate effective goal regulation. Conducive affordances include opportunity for immediate and concrete engagement in active groups that are powered by conspiracy narratives, infused with cosmic significance, encouraging of moral violence, and sealed with religious unfalsifiability. We propose that ARR is rewarding because it can spur approach motivated states that mask vulnerability for people whose dispositions and circumstances would otherwise leave them mired in anxious distress.

## Introduction

“We have killed all of the children in the auditorium…what do we do now?”

—Taliban gunman, December 16, 2014

After methodically shooting all 132 children and 12 teachers at a school in Peshawar, Pakistan, the Taliban militant, Abuzar, called his handler for further instructions. The answer came back, “wait for the army to arrive, kill them, and then blow yourself up” (Khan, 2014). Abuzar and the six other gunmen complied and detonated their vests on cue. This kind of self-immolating, aggressive religious radicalization (ARR) has recently animated high profile Islamist atrocities by the Islamic State (IS), al-Qaeda, Taliban, Boko Harem, al-Shabaab, and lone-wolf extremists all over the world. Although Islam is currently in the spotlight, ARR is not a Muslim phenomenon. Throughout history its callous extremes have blighted all major religions traditions (Armstrong, 2000). This paper provides a theoretical framework with data-driven hypotheses about how basic human motivations interact with situational affordances to make ARR alluring.

In the recent IS instantiation, over 30,000 young men and women from over 100 Asian, Middle Eastern, and Western countries have abandoned their normal lives to go fight with IS in foreign territory and abject living conditions. Indeed, more British Muslims have joined IS than the British military (Barrett, 2014a,b; BBC, 2015; The Soufan Group, 2015; Weaver, 2015). When they get there they sometimes burn their passports as a show of commitment before submitting to the harsh regimen of discipline for the fascist cause. They know they may die soon after arriving, and many do, either as suicide bombers or as casualties in battles picked with a more powerful enemy—a coalition that includes many of the world’s most powerful countries. Despite the shocking atrocities perpetrated by IS that include beheadings, crucifixions, rapes, pedophilia, and genocidal slaughter (BBC, 2014), and that have been well publicized on YouTube, IS continues to attract foreign recruits who are often among the most zealous (Barrett, 2014b). What is the appeal of enthusiastically perpetrating atrocities in the name of a religion that preaches mercy? How can personality and demographic profiles of recruits so often be normal and well-educated (Post, 1990, 2005; Barrett, 2014a).

We present a basic motivational framework that contests popular views claiming ARR is primarily a pragmatic revolutionary strategy, or driven by self-serving superstitions, or fueled by the particularly aggressive nature of any particular religion. We draw on classic and contemporary psychological theory and over 30 years of experimental research showing how and why psychological threats cause belligerent defenses and reactive approach motivation (RAM; McGregor et al., 2010a; Jonas et al., 2014). We hypothesize that ARR arises from personal predispositions, anxiogenic threats, and group affordances that combine to divert normal motivational processes toward approach motivation for ARR. Each factor alone is not enough, but conducive personality, threats, and affordances, together, are potent. Our view is compatible with insights about how motivation for worldview defenses, significance, meaningful engagement, identity-fusion, group-based control, belonging in action groups, and compensatory conviction and consensus might propel ARR (McGregor et al., 2001, 2005; Rothschild et al., 2009; Atran, 2010; Sageman, 2011; Fritsche et al., 2013; Hogg, 2014; Kruglanski et al., 2014; Swann and Buhrmester, 2015). Our view augments this previous work by grounding its hypotheses in primitive motivational substrates that can provide additional depth to the emerging understanding of motivation for ARR.

Our main premise is that ARR is rewarding because it spurs approach motivated states that mute anxiety for people whose personalities and social circumstances would otherwise leave them anxious and depressed (McGregor et al., 2010b; Jonas et al., 2014; Hayes et al., 2015). We begin by reviewing classic theories foundational to understanding ARR. We then link them to contemporary goal-regulation premises, theories, and recent advances in RAM theory. We next define the components of ARR, and use empirical research findings to justify our personality × threat × affordance hypotheses. We conclude by suggesting strategies for testing our hypotheses in the lab and real world.

## Classic Theory

A premise in classic and contemporary theory is that conflicts, frustrations, and uncertainties can propel belligerent reactions far removed from the eliciting difficulties. Sigmund and Anna Freud popularized the view that conflicting motivations in competing directions can arouse bizarre and extreme defenses against the ensuing anxiety. “Excessively intense,” “supervalent” thoughts form “mental dams” that effectively repress the offending conflicts Gay (1989, pp. 200, 261–262). From this perspective ARR-relevant defense mechanisms such as turning against the self, rationalization, fantasy, regression to childish tendencies, and projection of one’s own hostilities onto others would be considered mechanisms of repression that help people escape from other motivational conflicts in their lives (Freud, 1946).

Lewin (1933) brought Freudian ideas into a more general theory of motivation and goal regulation by showing that conflicts and uncertainties cause a kind of anxious tension that persists as long as goals remain impeded. If people have no clear way to relieve the tension arising from their (often social) conflicts then they escape from the field of tension by resorting to fantasy, submission, or belligerence (Lewin, 1933, 1935).

Neo-analytic theorists similarly proposed that aggression is a reflexive response to frustration for people and animals. The frustration-aggression hypothesis (Dollard et al., 1939; Berkowitz, 1989) is implicit in neoanalytic views that failure to forge identities grounded in prosociality tilts people toward fanaticism and oppositional power over others (Horney, 1950; Ansbacher and Ansbacher, 1956; Erikson, 1959). Horney (1950, pp. 86–109, 184) and Adler (Ansbacher and Ansbacher, 1956, pp. 259–261) respectively referred to this tendency as “arrogant righteousness” and “neurotic pride” or a “superiority complex,” wherein grandiose and oppositional facades mask insecure selves. Fromm (1941) viewed such defensive tendencies as escapes from existential freedom and saw conformity, authoritarianism, fascism, and destructiveness as symptoms of human’s inability to cope with the frustrating uncertainty about how to make choices in life. People cling aggressively to externally referenced “frames of orientation and devotion” to avoid becoming overwhelmed by existential uncertainty (Fromm, 1947, p. 48). Antisocial extremes replace gnawing uncertainty with decisive commitment that relieves angst. Durkheim (1897/1951) viewed uncertainty about what to do as noxious enough to cause suicide. If viable family relationships or cultural norms are not available to provide clear direction and purpose, people use suicide to escape the unbearable burden of choice (Durkheim, 1897/1951; see also Baumeister, 1990).

Sartre’s (1956) existential philosophical perspective similarly held that uncertainty arising from radical freedom spurs attempts to escape from the nausea of uncertain self-awareness by conforming to group norms (Barnes, 1973). Under this view, masochistic extremes of submission to authority and sadistic extremes of domination and hate are attempts to suppress uncertainty arising from absence of objective truths that could guide one’s choices in life. Masochism and sadism reduce the uncertainty associated with awareness of multiple perspectives by eliminating one’s own and/or others’ subjective perspectives. Problematic subjectivity, with its conflicting perspectives and uncertainties, is replaced by the solid ground of supposed objective authority.

These neoanalytic and existential ideas were the foundation for the Adorno et al. (1950) treatise on the authoritarian personality and causes of fascist disdain for deviants. Authentic identity development requires a vulnerable process of trial and error exploration (Rogers, 1951; Erikson, 1959; Marcia, 1980; Deci and Ryan, 1991). If this process is blocked by unsupportive people or chaotic social structures, then confident personal values and priorities for resolving uncertainty are not developed, and people turn to the dictates of powerful others and groups. Fusing or identifying with a fascist ingroup can thereby become psychologically vital, and critics become the enemy.

These classic theoretical views predict the link between conflict, uncertainty, and frustration, and aggressive radicalization, but they do not imply a specifically religious form of aggressive radicalization. James (2006) made that connection around the time that Freud was penning his first ideas about repression via commitment to excessively intense thoughts (Gay, 1989, pp. 200, 261–262). James (2006, p. 240, 399) observed that “religious rapture and moral enthusiasm are unifying states of mind that incline the sand and grit of selfhood to disappear,” and that can unify a “discordant self.” Consistent with our approach-motivation hypothesis he further proposed: “We shall see how infinitely passionate a thing religion at its highest flights can be. Like love, like wrath, like hope, ambition, jealousy, like every other instinctive eagerness and impulse, it adds... a new sphere of power” (James, 2006, p. 58).

These repression-related views were first put into goal-regulation language by Lewin’s (1933, 1935) understanding of how strong goals and commitments can clear away other conflicts, leaving people feeling sanguine and single-minded (reviewed in McGregor, 2003). Lewin (1933, p. 609) paved the way for a goal-regulation view of zealous religious devotion as an idealistic commitment that can function like a motivational “field of force” to push other uncertainties and frustrations out of awareness (see McGregor et al., 2010b, 2012b, for elaboration on the goal and emotion-regulation function of ideals). Contemporary social psychological and social neuroscience research now provides a clearer, less metaphorical understanding of the basic motivational mechanics beneath Lewin’s seminal goal-regulation ideas.

## Contemporary Theories of Threat and Defense

In the second half of the 20th century, personality and social psychological research began empirically testing and refining classic theories about causes of defensive social phenomena related to ARR. Hundreds of experiments on cognitive dissonance theory (begun by Lewin’s student, Festinger, 1957) demonstrated that experimentally manipulated cognitive conflicts could cause extremes of opinion rationalization that defied logic, including dubious claims by religious cult members (Festinger et al., 1956). The first generation of dissonance theory and research had its roots in classic psychodynamic theory, from Freud, through Lewin, to Festinger’s experimental demonstrations. Its growth became even more psychodynamic with neo-analytically inspired demonstrations of compensation. Research began to support Allport’s (1943, p. 466) assertion that various ego defenses provide “fluid compensation” for psychological discomfort arising from threats, conflicts, and uncertainties. Self-serving affirmations of worth, conviction, morality, meaning, or adaptive adequacy of any kind were found to mute threat-induced distress, even if they did not directly address the content of the original threat (e.g., see Steele, 1988; Tesser, 2000; McGregor et al., 2001; Heine et al., 2006). As Lewin would have predicted, the affirmations make the anxious uncertainties and conflicts less motivationally salient (McGregor, 2006a), which makes them less aversive (McGregor et al., 1999).

A proliferation of threat and defense theories sprouted around the intuitive idea that fluid compensation occurred because threats to a psychological resource (esteem, security, integrity, immortality, belongingness, meaning, or control) aroused compensatory reactions that served to replenish the threatened psychological resource, often in disguised ways. For example, if an anxiety-inducing failure threat caused a worldview defense reaction involving hostile derogation of an outgroup or moral offender, various compensatory theories would interpret this as disguised compensation for threatened esteem, certainty, conviction, integrity, immortality, security, belongingness, meaning, or control (i.e., depending on the authors’ theoretical allegiance; see Jonas et al., 2014 for review). Compensation perspectives would accordingly view ARR as an indirect strategy for restoring whatever basic need had been indirectly undermined by psychological threat. As powerful and generative as resource compensation theories became, their proliferation and explanatory competition ushered in an integrative theoretical approach to understanding threat and defense processes at a more basic motivational level.

## Goal Regulation Theory: A Lens for Understanding ARR

Primitive motivational structures in humans, mice, and other vertebrate brains are organized around goal dynamics and anxiety. Behavioral, lesion, and pharmacological studies reveal that goal frustration and uncertainty are the prime causes of anxious distress, mediated by the septo-hippocampal behavioral inhibition system (BIS; Gray and McNaughton, 2000). The neurophysiology of anxiety is different from that arising from other aversive states like sadness or panic. As with Lewin’s (1933, 1935) idea of tension, anxiety arises from the approach-avoidance conflicts inherent in goal blockage, uncertainty, novelty, and frustration. Recent revisions to Gray’s theory (by his student, Corr, 2008), emphasize that BIS activity is inversely related to activity of the other main motivational system, the behavioral activation system (BAS). The BAS promotes single-minded approach motivation—the “impulse to go toward” (Harmon-Jones et al., 2013, p. 291). It is an eager, goal commitment system that, when active, mutes the BIS (Corr, 2008; Nash et al., 2012). Effective goal regulation is maintained by the interplay between these two systems. When goals are going well the BAS predominates and makes people feel energized, eager, and single-mindedly committed to approach of the focal goal. BAS activation automatically inhibits possible distractions and conflicts, and allows people to focus on eagerly approaching identified incentives, free from anxious preoccupation with their worries. If a serious conflict or uncertainty erupts along the way and succeeds in activating the BIS, however, then the BAS is muted and three primary BIS outputs result. All ongoing goals are inhibited to stop the animal from doing whatever is not working; anxious distress further discourages persistence at ongoing goals; and vigilance dilates to help the animal notice a wider range of possible threats or opportunities that could cue single-minded withdrawal or approach to get the animal out of the conflicted and anxious state, and onto a more viable track.

This account of BIS and BAS follows Lewin’s (1933, 1935) view of how tensions aroused by goal conflict can be blocked out by focused immersion in other goals. In more contemporary language, activating the BAS through goal commitment eliminates the hesitant, anxious vigilance associated with the BIS. Throwing oneself into a commitment like ARR could accordingly be a way to effectively repress other anxious conflicts (Harmon-Jones et al., 2009; Nash et al., 2011; McGregor et al., 2013b; see Jonas et al., 2014 for overview of the underlying basic processes and links to threats and defensive reactions).

This goal and emotion regulation function of the BAS has been empirically demonstrated in humans by personality and social psychology experiments focused on goal commitment, shielding, and implementation. Tenacious goal commitments and related eager approach motivation processes activate the BAS and narrow motivational attention to the focal goal (Harmon-Jones et al., 2011, 2012), shielding it from interference by other conflicting or competing goals (Shah et al., 2002). The single-mindedness facilitates vigorous goal completion (Elliot and McGregor, 1999; Harmon-Jones et al., 2009) and is also affectively rewarding—people feel more optimistic when actively engaged in implementing a commitment than when deliberating about alternative possibilities (Taylor and Gollwitzer, 1995). The same insulation from conflicts and uncertainties can come from eager immersion in more abstract goals, values, and group identifications (McGregor et al., 2001; McGregor, 2006a, Study 1; McGregor and Marigold, 2003, Study 4; McGregor et al., 2005, Study 4) because values and groups also activate the BAS (Agroskin, unpublished doctoral thesis, McGregor, unpublished data), especially if they are extreme (Sleegers et al., 2015). Cybernetic theories of goal regulation posit that ideals and values are abstract goals that function as self-guides or system concepts to organize and coordinate the array of subordinate, more concrete goals (Higgins, 1996; Carver and Scheier, 1998; Hirsh and Kang, 2015). Extremes are resistant to ambivalence, so they should be particularly effective as clear self-guides (Newby-Clark et al., 2002).

These basic goal regulation processes furnish a parsimonious, mechanistic account for the operation of classic theoretical ideas about why people in anxious circumstances turn to excessively intense thoughts, moral enthusiasms, exclusive fields of force associated with goals, or the other manifestations of rigid and extreme opinion, devotion, and authoritarian hostility. All might effectively function as levers for BAS-activated approach-motivated states that are rewarding because they mute BIS activity. Considering that anger is also a powerfully BAS-activating phenomenon (Carver and Harmon-Jones, 2009), it seems plausible that extreme commitment to ARR might be an appealing response to anxiety-inducing threats, uncertainties, frustrations, and injustices because ARR contains several elements capable of transitioning people from “anxiety to approach” (Jonas et al., 2014).

## From Anxiety to Reactive Approach Motivation for ARR

The basic-process ideas outlined above were first organized in papers identifying exaggerated conviction, pride, consensus, and intergroup animosity as levers for approach-motivation-related states that people use to downregulate threat-activated BIS (McGregor et al., 2005; McGregor, 2006b). Over the last 10 years this speculation has been empirically supported by rigorous experimental research. Anxiety-related threats (dissonance, uncertainty, failure, control loss, mortality salience, relationship distress, insecurity, goal-frustration) that have caused extreme “compensatory” reactions in past research have also now been found to cause neural indicators of BIS activation at first, and then RAM (reviewed in Proulx et al., 2012; Jonas et al., 2014). The measures of RAM include basic neural, perceptual, and affective evidence, along with eager and idealistic commitment to personal goals and commitments in everyday life (McGregor et al., 2007, 2009a, 2010a, 2013b; Nash et al., 2011; Greenaway et al., 2015). Importantly, these same threats also cause self-reported endorsement of religious extremes and increased willingness to kill and die for religious beliefs (Pyszczynski et al., 2006; McGregor et al., 2008, 2010b, 2013b; Rothschild et al., 2009; Wichman, 2010). Further, laboratory experiments now indicate that elements of ARR, and religious devotion itself, can cause neural, perceptual, and self-report evidence of approach motivation (reviewed in Jonas et al., 2014; Agroskin, unpublished doctoral thesis, McGregor, unpublished data). Anxiety-to-approach dynamics are thus well positioned to help explain the enigma of ARR.

### Pragmatic and Palliative RAM

It is important to acknowledge, however, that levers for activating RAM are not necessarily defensive and irrational. Constructive responses to anxiogenic circumstances can also provide RAM relief from anxiety. Indeed, this may be the most usual and adaptive function of anxiety-to-approach processes, as in tenacious striving for financial security after deprivation, or for success or love after failure or rejection. It is when direct resolution opportunities seem blocked and hopeless, however, that people turn to merely palliative defenses like ARR to activate RAM for relief.

It has been argued that ARR might be a direct, constructive form of freedom fighting aimed at restoring social justice for oppressed people (Armstrong, 2014). The suffering inflicted on Muslims caused by the long history of Western hegemony, hypocrisy, political interference, exploitation for oil, economic sanctions that mostly harm poor civilians, and military invasions have been identified as catalysts for Islamist extremes. Although realistic grievance is surely part of the story (Armstrong, 2014; Barrett, 2014a,b), and ARR may be partly motivated by pragmatic motivation to make constructive improvements, characteristics of ARR cast doubt on the adequacy of such straightforward explanations.

First, ARR is often rash and counter-productive. In one of the first examples of ARR that gave rise to the word “zeal,” an oppressed Zealot sect of Judaism assassinated anyone who disagreed with their extreme agenda, even those in their own group who did not seem devout enough. Their extremism brought annihilation from the governing Romans. A similar plight befell the first Christian Crusaders. After Pope Urban’s rousing 1086 CE speech about restoring the glory of Charlemagne and saving the Holy Land from Evil, a band of overly enthusiastic and unprepared Crusaders broke from France for Jerusalem before the designated date. In their zeal they began slaughtering anyone along the way who seemed a different race or religion. They were soon annihilated by the first wave of organized resistance that they faced (Durant, 1950). The violence of IS and other ARR groups seems similarly rash and counter-productive (Post, 1990; Barrett, 2014b). Their self-publicized atrocities may have some strategic value insofar as they discourage resistance in the towns they occupy, but they have also turned most of the world against them. Even predominantly Muslim countries like Iran, Iraq, Jordan, Saudi Arabia, Bahrain, United Arab Emirates, and Turkey, that might otherwise have been sympathetic to legitimate social justice grievances, have joined a coalition with Western powers against IS. The Muslim-on-Muslim atrocities of IS have even alienated the radical Islamist group, al-Qaeda, from which IS evolved (Barrett, 2014b).

A second reason for doubting the pragmatics of ARR is the retrograde nature of its religious claims. Mature religion is usually associated with humility, recognition of mystery, compassionate concern for others, and aversion to violence (Armstrong, 2006, 2009; Schumann et al., 2014; Shariff et al., 2015). These characteristics are at odds with the fascist, black and white, superstitious, and hostile characteristics of ARR that more closely resemble psychological defenses (Post, 1990; Jonas et al., 2014). Indeed, among ARR recruits there is often only a thin veneer of religious knowledge supporting their devotion (Atran, 2010; Barrett, 2014a). Pragmatic devotion would presumably be built on a more mature foundation.

The third reason to doubt the pragmatics of ARR is the risky self-destructiveness of its members. New recruits leave lives and loved ones behind and risk everything. A longer life of careful, strategic devotion to a solid cause would presumably accomplish more than a quick and dirty death for a dubious and sensational cause. The relish to join, fight, and risk for an extreme cause seems to have more psychological appeal than instrumental benefit (Nash, unpublished doctoral dissertation; McGregor et al., 2013b; Black et al., 2014; Hogg, 2014).

### A Concrete and Social Approach

Our specific hypotheses below about the kinds of people and circumstances conducive to ARR are informed by a recently advanced taxonomy of phenomena people approach for relief from BIS-activation. People react to anxiety-inducing experiences by approaching phenomena that are either personal or social and either concrete or abstract. The four domains of phenomena people use to activate RAM are accordingly concrete personal (e.g., money, personal aggression, power, control); concrete social (e.g., group membership, group aggression, group power, group status); abstract personal (personal values, ideals, moral convictions); and abstract social (collective worldviews, ideologies). Phenomena in all quadrants can be eagerly pursued to activate approach motivated states (Jonas et al., 2014). Ostensibly religious phenomena can populate all four quadrants, e.g., ritual action in the concrete personal quadrant; coordinated group rituals, coalitional action, or intergroup hostility in the concrete social quadrant; idiosyncratic ideals and values in the abstract personal quadrant; and consensual worship of cultural symbols, worldviews, and meanings in the abstract social quadrant. Our working definition of religion emphasizes the idealistic aspects of religious devotion (see below) but the concrete aspects can serve as accessible sacraments for orienting toward the idealistic elements, especially (as we develop below) for people who may prefer concrete engagement. Our view is consistent with Armstrong’s (2000, 2009) claim that fundamentalist ARR cleaves to the concrete in an arguably blasphemous attempt to remove the inherent mystery from its understanding of God. Our position here on the concrete nature of ARR is accordingly a departure from our past focus on more abstract aspects of idealistic religious devotion (e.g., McGregor et al., 2010b, 2012a).

Past investigations of phenomena people spontaneously use to activate RAM focused on abstract-personal levers. Random assignment to various, 2–5 min anxiety-related experiences (e.g., personal uncertainties, mortality salience, relationship insecurities, social exclusion, performance anxiety) caused people to become more extreme in their moral opinion conviction and consensus estimates, and in pursuit of idealistic goals, convictions, and meanings (McGregor et al., 2009b). These abstract-personal reactions appear to relieve anxious distress by activating approach-motivated states (Jonas et al., 2014). The same threats also heighten abstract-personal varieties of religious devotion—more confident certainty in the objective truth of self-identified religious beliefs, more determination to live according to them, more identification with them, and more willingness to argue in defense of them (McGregor et al., 2008, 2010b, 2013b).

People with eager, idealistic, and approach-motivation-correlated traits have been most inclined toward use of abstract-personal levers for RAM (reviewed in Jonas et al., 2014). Based on this we had initially assumed that eager, idealistic, and confident kinds of people should be especially inclined toward ARR, under the assumption that ARR was essentially an abstract ideology (McGregor et al., 2008, 2010b, found that eager, idealistic people were most inclined toward reactive, abstract religious extremes). Our most recent research, however, suggests that the kind of abstract-personal religious zeal that our previous research focused on is *not* what ARR usually revolves around. Like ethnocentrism and outgroup derogation, ARR is usually idealistically impoverished, and seems to revolve more around opportunity for concrete participation in hostile authoritarian groups that mobilize aggressive action disguised by a veneer of oversimplified ideology (Post, 2005). We propose that it is through this concrete participation in simplified, black and white ideologies that identity-weak people (i.e., morally bewildered) are able to acquire an externally referenced sense of “identity, purpose, belonging or spiritual fulfillment” for a “greater sense of purpose and meaning in their lives” (Barrett, 2014a, p. 18; Barrett, 2014b, p. 7; Atran, 2015). Groups, aggression, and action can activate approach motivate states, and approach motivated states feel meaningful (McGregor et al., 2012b). Given the seeming concrete and authoritarian characteristics of ARR, we here hypothesize different predisposing personality traits than in our past research. Whereas identity-strong people have tended toward abstract-personal levers for RAM, identity-weak people should be most inclined toward ARR.

Without the guiding and constraining influence of mature personal or religious identity, which tend to be prosocial, the more concrete, risky, aggressive, hostile, and coalitional kinds of levers for activating RAM could be especially alluring. Indeed, markers of concrete coalitional but not intrinsic religion predict support for suicide bombers and prejudice (Allport and Ross, 1967; Ginges et al., 2009). A limitation of concrete, extrinsic religion, however, may be that it is less reliable and efficient in maintaining approach-motivated relief. It requires involved physical engagement because it may not be as easily summoned as abstract ideals in private imaginations (McGregor et al., 2012b). Further, despite temporary relief engagement in ARR might provide, its antisocial hostility presumably impedes harmonious social functioning and brings more social conflict and anxious insecurity over time. Indeed, internal conflict is predicted to be the downfall of IS (Barrett, 2015).

Once it becomes clear to identity-weak people that ARR doesn’t work as well as hoped, they might either amplify zeal, or withdraw altogether from life so as to activate unmitigated avoidance motivation. Anxiety is a function of the BIS response to simultaneous approach and avoidance cues. It can accordingly be relieved by either singular approach or singular avoidance (Hayes et al., 2015). Withdrawal from concern with life outcomes would make hostile and antisocial levers for RAM easier to engage without regard for possible consequences. In sum, the appeal of ARR may be that it offers opportunity for toggling between concrete approach (aggressive, powerful, hostile, coalitional) and fatalistic withdrawal from life through self-immolating extremes. Both are anxiety-relief strategies available to identity-weak people.

## Components of ARR

### Aggressive

By aggressive we mean a tendency to assert ones will against others, oneself, or any symbolic or concrete target in a way that can augment the feeling of power, status, or control vis-à-vis the target. Belittling, overpowering, or destroying others, the self, institutions, or properties are aggressive by this definition. Violence is a concrete manifestation of aggression.

### Religious

As reviewed above, philosophical and goal-regulation perspectives propose that humans need moral ideals to guide concrete goals. Without moral ideals humans can become mired in conflict among all the imaginable possibilities for action. Following Fromm’s (1973, p. 260–261) view that a primary existential need for humans is “an object of total devotion…to be a focal point of all his [sic] strivings,” we view religion as a vehicle for moral values that is often bolstered by consensual rituals and symbols (Geertz and Banton, 1966). This morality-focused view of religion is compatible with Durkheim’s (1976/1912) seminal claim that religions are not necessarily about gods (though they often are—Gods are potent symbols), but rather that they revolve around group convictions about what is to be valued as sacred, above any particular temporal concern. Worship and ritual involve active group affirmations of these sacred phenomena as worthy of veneration. From this perspective, the essence of religion is less about superstitious belief in existence of supernatural entities and their intra-group moral policing function (cf., Shariff and Norenzayan, 2007) than about eager devotion to moral commitments that function as arbiters for coordinating enthusiastic action within individuals and groups.

Some historians of religion have similarly concluded that the essence of religious devotion revolves around functional meanings that help people set priorities for effective living (Smith, 1986; Armstrong, 2006). Armstrong argues that the jagged evolution toward compassionate values across religious traditions has been occurring because prosocial values are the only kind that can sustain consensus and cooperation, and constrain costly inter-group violence. Drawing on existential, psychological, and religious-historical perspectives we accordingly define the essence of religion as a moral orientation toward action that is often but not necessarily anchored by ideas of God, that is often but not necessarily bolstered by consensual ideology and ritual, and that is usually prosocial but can sometimes endorse aggressive and fascist extremes.

The vulnerability toward fascist extremes exists because devotion to abstract moral ideals can be difficult for solitary individuals. Ideals and values have no concrete referents and so rely on consensus for confidence (Festinger, 1950; Wicklund and Gollwitzer, 1982; Hardin and Higgins, 1996). “Conservative,” coalitional moral foundations relating to ingroup consensus (authority, loyalty, purity) sometimes co-occur along with the more universal social justice moral foundations (fairness and care; see Graham et al., 2009 for evidence of conservative vs. universal moral foundations). Among people who are vulnerable to anxiety (Jost et al., 2003; Olvet and Hajcak, 2008) and in frustrating circumstances conducive to aggressive reactions and ingroup cohesion and consensus (Dollard et al., 1939; Shah et al., 1998; Kruglanski et al., 2002; Miller et al., 2003; McGregor et al., 2005; Burke et al., 2010), the balance between social justice idealism and the more concrete coalitional and authoritarian moral foundations can list toward coalitional/authoritarian. When they eclipse social justice, ARR can flourish.

### Radicalization

Radicalization is a shift from mainstream to anti-normative or comparatively extreme ideological convictions that animate eagerness to challenge the status quo. Radicalization is neither necessarily aggressive nor religious. Martin Luther King Jr. and Mahatma Ghandi were radicals, as was Tommy Douglass, the politician who won universal health coverage for Canadians in the 1960s. Barack Obama’s quest for more universal healthcare in the US is still considered radically subversive by half of the US population. All of the above might be considered religious radicals to the extent that their radical determination for social justice was girded by their own religious values. Their somewhat religious radicalization was pragmatic and constructive, however, not aggressive.

From our perspective ARR is usually motivated more by the psychological appeal of the radical and aggressive lifestyle than by a constructive assessment of what the radical agenda is likely to accomplish or by thoughtful religious integrity (see also Post, 1990; Atran, 2010, 2015, for more on the superficial religious knowledge and idealistic impoverishment of ARR recruits). We propose that people are drawn to ARR simply because it feels right. Here we develop a theory for why something that seems bizarre and abhorrent to most people could be attractive for some. Based on past theory and research we hypothesize that specific personality, threat, and affordance factors combine to make ARR feel right.

## Factors Conducive to ARR: Personality, Threat, and Affordance

When episodes of ARR occur they are typically met with astonished exclamations of “why him,” “why there.” Why, for example, did one sleepy Norwegian town spawn eight IS recruits (Higgins, 2015), one pre-university school in Montreal 11 (Perreaux, 2015), and one elementary school in Morocco five of the seven Madrid train bombers (Atran, 2010)? Part of the reason may be that the individuals belonged to tightly knit, action-oriented social networks, such as neighborhood groups or soccer teams that set a group-action template for them to engage in heroic adventure together in another context (Atran, 2010). But most people in buddy-groups are not drawn to ARR. Blaming personality predispositions or demographic characteristics is also little help because perpetrators of ARR are surprisingly normal (Post, 2005). Environmental threats, frustrations, and anxieties may similarly be ingredients (McGregor et al., 2010b), but most anxious and frustrated people do not turn to ARR.

Difficulty identifying causal factors in past research may have arisen from failure to simultaneously consider combined personality, threat, and affordance interactions. Here we identify 10 ARR-facilitating influences across the three factors (personality, threat, and affordance). For each influence to be above average in prevalence would occur by chance with a 2^10^ probability of only 1/1024. It is not surprising, then, if our view is correct, that isolated personality traits or threat influences often fail empirical tests of relevance. Some laboratory studies (described below) have found combinations of a few of these 10 influences can cause self-reported movement toward aspects of ARR. Real life ARR surely requires more influences to align, however, because barriers to ARR in real life are higher than for self-reported opinions or intentions.

The personality factor includes three influences from normal-range personality traits that lean toward: (a) oppositional personality traits related to aggression (disagreeableness, hostility, anger, narcissism, or low self-control); (b) high BIS personality traits that incline people toward the experience of anxiety; and (c) identity-weak personality traits that undermine capacity to assert personal value priorities, take initiative, or maintain self-regulatory control. The threat factor includes two influences: (a) external control threats that undermine faith in the social system (that identity-weak people are especially inclined to rely on; and (b) life circumstances that underscore hopelessness. The affordance factor includes five influences: (a) opportunity for immediate, concrete engagement with active ARR groups, (b) a consensual injustice narrative that condenses conspiracy-theory blame for system and self-dissatisfaction onto the outgroup, (c) narratives that convey heroic participation in a cosmic battle between good (own group) vs. evil (outgroup), (d) justifying narratives for religious aggression, and (e) unfalsifiable religious arguments.

### Personality

#### Oppositional

A history of delinquency or criminal activity prior to ARR is a common but not a clear predictor (Post, 1990; Atran, 2010). Its incidence may arise from intercorrelated dispositional leanings toward aggression, narcissism, disagreeableness, and low self-control that have been linked to ARR-related phenomena in other research (and that are all correlated at around *r* = 0.3, McGregor, unpublished data). Trait-aggressive and narcissistic people are especially receptive to aggressive media exposure and inclined toward displaced hostile reactions to frustration, perceived provocation, and rejection (Bushman, 1995; Bushman and Baumeister, 1998; Anderson and Dill, 2000; Twenge and Campbell, 2003). Narcissistic and the other “dark tetrad” traits of psychopathy Machiavellianism and sadism (Paulhus, 2014) are significantly correlated with each other and with high agency, low agreeableness, punitiveness, and callous low empathy (Watson et al., 1984; Campbell et al., 2002; Paulhus and Williams, 2002; Vernon et al., 2008; Jones and Paulhus, 2010; McGregor et al., 2013a). Aggressive reactions to threats are approach-motivated (Carver and Harmon-Jones, 2009), and should accordingly focus one on tenacious personal goals with diminished regard for others’ perspectives (Hogeveen et al., 2014; Sassenrath et al., 2014). Indeed, people with low agreeableness scores are mistrusting, devious, selfish, stubborn, arrogant, and callous (Costa and McCrae, 1991). Low self-control is also associated with delinquency, criminal behavior, physical and verbal aggression, self-directed aggression, and extreme and risky reactions to various environmental threats (Tagney et al., 2004; Nash, unpublished data; see also links with low conscientiousness and the dark personality traits, Jakobwitz and Egan, 2006). High scores on these intercorrelated and highly heritable oppositional traits should accordingly predispose people to the appeal of ARR, if other traits, threats, and affordances are also conducive (Costa and McCrae, 1991; Miles and Carey, 1997; Baker et al., 2008; Vernon et al., 2008; Beaver et al., 2009).

It is important to emphasize that these predisposing tendencies need not be in the abnormal range. The vast majority of ARR perpetrators have normal range traits. Our hypothesis is that even normal range tendencies should be enough to combine with the other factors to make ARR appealing. There are also several different kinds of ARR for which the conducive personality traits are likely differentially important. For example, lone-wolf perpetrators and leaders of ARR movements should be most likely to score highly on oppositional traits. In contrast, the foot soldiers and joiners of established movements may be less likely to be oppositional and social dominance oriented, and are more likely to be submissive authoritarians who participate in the oppositional tendencies by association (Son Hing et al., 2007).

#### Anxious

By anxious traits we mean those associated with a predisposition toward BIS activity that may or may not be reflected in consciously self-reported state anxiety (which can be attenuated by defenses and which is often out of sync with physiological indicators). The BIS generates early signals conducive to anxiety, vigilance, and caution in uncertain or conflict-laden circumstances, and people with anxious traits are more inclined than others toward these responses (Hirsh and Inzlicht, 2008; Proulx et al., 2012). Correlational studies show reliable links between anxious and aggressive traits (e.g., *r*s of 0.4 and 0.5 between neuroticism and measures of aggression and hostility in our recent sample of 299 culturally diverse college students; McGregor, unpublished data; see also Jakobwitz and Egan, 2006).

Anxious arousal also mediates defensive reactivity. After threats, if participants can misattribute their anxious arousal to a mundane external cause (e.g., a placebo, or uncomfortable room) they no longer react defensively to threats by rationalizing or becoming extreme (reviewed in Jonas et al., 2014). Anxiety-related need for structure also mediates the effect of threats on worldview defense (Agroskin and Jonas, 2013). Anxiety-related traits and states including felt uncertainty, trait neuroticism, uncertainty-aversion, need for structure, and sense of victimhood, also moderate defensively extreme lifestyle choice, worldview defense, violence, and religious zeal reactions to uncertainty, mortality, control deprivation, and relationship insecurity threats (Hirschberger et al., 2009; Juhl and Routledge, 2010; McGregor et al., 2010b, 2013b; Agroskin, unpublished doctoral thesis).

The interconnected and anxiety-linked constructs of low implicit self-esteem and relationship attachment insecurity (DeHart et al., 2006) similarly moderate distressed, aggressive, extreme, and worldview zealous reactions to failure, relationship, insecurity, and mortality threats (Mikulincer and Florian, 2000; McGregor and Marigold, 2003; Jordan et al., 2005; McGregor et al., 2005; McGregor and Jordan, 2007; Schmeichel et al., 2009; Laurin et al., 2014; Nash et al., 2014).

Recent advances in techniques for indirect assessment of *states* related to anxiety have also begun to implicate anxious distress in ARR reactions. A wide variety of threats that have caused ARR-related defenses in past research only inconsistently arouse self-reported anxiety. However, almost all of them have been shown to heighten electrical activity source-localized to the anterior cingulate cortex of the brain which has been associated with anxious distress and BIS activation (Proulx et al., 2012). These same threats also elevate self-reported anxious distress that is delayed or retrospective, presumably because delay or retrospection evades the defenses that can obscure self-reports of anxious distress immediately after threats (McGregor et al., 2001; Nash et al., 2011; Agroskin, unpublished doctoral thesis). Taken together in light of recent RAM theorizing (Jonas et al., 2014), the links between ARR-related phenomena and BIS-related states and traits suggest that anxious traits related to high BIS activity should predispose people towards using ARR to mask their distress.

High BIS personalities may also be drawn to ARR because of their discomfort with abstraction and their attraction to concrete engagement in low-level thoughts and goals. High BIS personalities feel energized and mobilized by immersion in details of concrete action and are averse to focus on abstract reasons for “why” which can cue distressing rumination. Indeed, concreteness manipulations (e.g., being randomly assigned to write about the “how” vs. “why” of various goals) not only relieve distress after anxious experiences (Watkins et al., 2008) but also causes high BIS participants to run harder and burn more calories on a treadmill endurance test, squeeze with more tenacity on a hand-gripper, persist to better performance in a speeded data-entry task, report higher eager excitement on a self-report questionnaire, to feel more optimism about personal goals, and show an increase in left frontal brain activity, characteristic of approach motivation (Tran et al., unpublished manuscript).

High BIS personalities may prefer the clarity of concrete action to the ambiguity of abstraction because focus on concrete steps regarding what to do alleviates the potentially bewildering tangle of uncertainties about values and identity, especially for identity-weak people (Vallacher and Wegner, 1989; Baumeister, 1991). Developmentally, however, patience with uncertain abstraction may be required if one is to identify, simulate, and hone reliable personal values to identify with (Adorno et al., 1950; Erikson, 1959; Marcia, 1980). Values need to be test-driven in comparison with other existential options before they can be adopted as an autonomous and intrinsically motivating part of personal identity (Ryan and Deci, 2000; La Guardia, 2009). This uncertain discovery process is more likely to feel threatening for people with high BIS reactivity whose anxiety circuits are most easily overloaded by uncertainty (Hirsh and Inzlicht, 2008). For them, defenses that allow escape from anxious conflicts by either unmitigated approach or avoidance motivation would be appealing (cf; Marcia, 1980; Jonas et al., 2014; Hayes et al., 2015).

When anxious people choose the approach route, they should accordingly be especially drawn to concrete and immediate commitments that limit exposure to the anxious burdens of abstract selfhood (Baumeister, 1986). They should also be drawn to the fascist structure of ARR groups that can provide direction and a sense of meaning without requiring self-clarity. Indeed, when faced with mortality reminders, mildly depressed people were most likely to bolster meaning with jingoistic judgments (Simon et al., 1998). Concrete and active engagement with fascist groups should accordingly hit the motivational sweet spot for anxious people by allowing them to restore approach motivated states without having to think abstractly.

#### Identity-Weak

At any choice point, people can imagine multiple possibilities for action. Classic and contemporary theories of human choice and goal regulation hold that identity-strong people constrain the potential for uncertainty and conflict among imagined alternatives by using idealistic abstractions (i.e., highest values) for guidance (reviewed in McGregor, 2004, 2006b, 2007). Committed identification with high values can help guide choices and thereby allow people to function with confidence and efficacy through frustrating or uncertain circumstances (Lydon and Zanna, 1990; Kroger and Marcia, 2011). Put another way, clarity about how to be can effectively guide what to do. Committed values function like abstract goals and can also activate approach motivation directly and further relieve anxious distress by mere reflection (McGregor et al., 2001, Study 1; Creswell et al., 2006; McGregor, 2006a; McGregor et al., 2012b). Indeed, for highly meaning-seeking people, even brief reflection on personal values activates approach motivated states (neural, perceptual, and self-report evidence in McGregor, unpublished data).

In contrast, identity-weak people who lack clear value identifications to guide action and relieve anxious distress are less able to cope constructively with frustrating circumstances. Indeed, identity-weak people (i.e., with low scores on a trait measure of idealism) reacted to mortality, control deprivation, failure, and relationship threats by becoming especially anxious, bewildered, and demotivated (McGregor and Marigold, 2003; Ferriday, unpublished master thesis; Prentice et al., unpublished data). Self-doubt also predicts materialistic reactions to uncertainty (Chang and Arkin, 2002). Moreover, when simultaneously confronted with multiple vulnerabilities and threats (life-dissatisfaction, goal frustration, mortality salience) identity-weak people become fatalistically withdrawn from personal goals, report being depressed, and report that they wish to live shorter lives (Hayes et al., 2015). In contrast, identity-strong people (i.e., high self-esteem, high scores on trait idealism) cope with anxious distress relatively easily by focusing on personal ideals about self-worth or value conviction that activate approach motivated states and thereby relieve the anxiety (Dodgson and Wood, 1998; McGregor and Marigold, 2003; McGregor, 2006a; McGregor et al., 2007, 2009a; Schmeichel et al., 2009; Nash et al., 2010; Schumann et al., 2014; see also McGregor et al., 2010a for evidence that experimentally priming ideals can also activate this process).

When high personal values (which are usually prosocial; Crocker et al., 2008; Schumann et al., 2014) are not available or salient, on the other hand, we propose that people will be more likely to revert to concrete, angry, controlling, and jingoistic foci. All can activate palliative approach motivation in a concrete way (Keltner et al., 2003; Carver and Harmon-Jones, 2009; McGregor, unpublished data). Participants with low scores on a dispositional idealism scale also reacted to social-order and relationship-security threats by becoming especially bewildered, and by becoming extremely devoted to the concrete and social (angry, group-related) aspects of religious zeal but not the abstract personal. After threat they rated their personal goals in life as being more random and out of control, and reported more willingness to support war and die for their religious beliefs, more allegiance to their own religious group, more hostility and less openness to people with different religious beliefs, more confidence that God would give them power and take care of them, and more willingness to go to extremes for God (Ferriday, unpublished master thesis).

Both high self-esteem and idealism are significantly correlated with each other and with other constructs related to personal agency (McGregor et al., 2007, Study 2). People with low scores on either self-esteem or idealism report significantly lower power, self-control, drive, hope, and efficacy, and higher anxiety, depression, and rumination (McGregor, unpublished data). The low personal agency arising from difficulty with ideals is consistent with classic and contemporary theories of the self-regulatory role of ideals and values. Accordingly, when people lack personal agency they cleave to sources of group-based and external control, through heightened allegiance to active groups, external agencies, and religious authority (Fritsche et al., 2008, 2011, 2013; Kay et al., 2010; Landau et al., 2015; Stollberg et al., 2015).

On the surface it may seem perverse to argue that aggressive religious radicals are identity-weak when their rhetoric brims with moral certainty and megalomanic conviction. Theories of narcissism, however, hold that such overt entitlement and importance is a reaction to weak capacity for nuanced and viable ideals. The rigid narcissistic shell shields against anxious distress of a vulnerable and lost soul (Kohut, 1971; Kernberg, 1975; see also Kroger and Marcia, 2011, for related research on identity foreclosure). Indeed, reactive narcissistic rage, punitiveness, and callous disregard for others is especially prevalent among people whose entitled grandiosity is belied by vulnerability and shame (McGregor et al., 2005, 2013a; Krizan and Johar, 2015). From this perspective, ARR is a kind of narcissistic response that appeals to identity-weak people. They should be most vulnerable to the dubious and grandiose religious ideals that animate ARR because they have no clear opposing identity and are most in need of bolstering and escaping the problematic self. Accordingly, they should be most inclined both toward extremes of bolstering the self by joining ARR groups (Fritsche et al., 2013; Swann and Buhrmester, 2015), and de-individuated escaping of the self by cleaving to group authority (Postmes and Spears, 1998). Both processes would orient them toward group loyalty and angry, concrete action without pangs of conscience.

In sum, clear values provide resilience in the face of anxious distress. They aid instrumental coping by providing agile capacity for assessing, prioritizing, and adjusting goals. They also provide a ready focus for activating approach motivation and relieving anxiety. They thereby help people cope with life’s frustrations, choices, and uncertainties in instrumental and palliative ways. Without authentic and confident personal value identifications, some other arbiter for making choices and soothing anxious distress is necessary. Rigid (concrete, literal) and extreme commitment to hostile, fascist, and authoritarian groups may be a default alternative for the dispositionally inclined.

### Threat

#### External Threat

For people like those just described who are dispositionally reliant on external sources of agency, threats that undermine external sources of agency should make ARR especially appealing. When personal control is weak (i.e., for anxious and identity-weak people) or unavailable, and a source of external agency (e.g., ingroup, god, government) is threatened, people should be inclined to switch allegiance to another seemingly viable source of external agency (Kay et al., 2008, 2010). Feeling excluded or alienated, or like one’s ingroup was unfairly treated or humiliated, or one’s country was out of control should make people with weak capacity for personal agency turn to other sources of external agency, such as ARR.

Preliminary support for this hypothesis comes from research showing that whereas high dispositional idealism predicts reactive *personal* agency (for personal goals and personal beliefs) after a personal agency threat (i.e., zeal for personal beliefs after a failure experience; McGregor et al., 2007, 2010b); low dispositional idealism predicts reactive allegiance to external religious agency after an external control threat (i.e., willingness to go to extremes for God and religious groups, and claims to derive strength and safety from a powerful God after threats to important relationships or to economic stability; Ferriday, unpublished master thesis). Based on these results and our extension of compensatory control and group-based control theories (Kay et al., 2010; Fritsche et al., 2013) we expect that because identity-weak people rely on external sources of agency in uncertain circumstances they should be especially bewildered and drawn to the external agency of ARR when their other external sources of agency are threatened.

Turning to ARR as a way to restore approach motivated relief from distress could be precipitated by various threats to institutional or relational sources of agency, such as war, sanctions, economic instability, high unemployment, system injustice, system incompetence, corruption, hypocrisy, relative deprivation, and cultural marginalization, ridicule, prejudice, ostracism, unfair social policies that relegate disadvantaged people to inescapable cycles of humiliation and hardship. Relational, domestic abuse, conflict, unfairness, boredom, or uncertainty could have a similar effect leaving people feeling overwhelmed and frustrated. External threats, whether societal or domestic should accordingly heighten the appeal of ARR. Consistent with this external-threat interpretation, the first three reasons suggested for the rise of IS relate to perceptions of systemic injustice against Muslims: (1) Shia (Iran, Iraq, Syria) oppression of Sunnis, (2) lack of confidence in governmental ability to protect social justice, and (3) the perception of a “Western-led onslaught” against Muslims by the West and their coalition (Barrett, 2014b).

#### No Hope

If the present is grim and frustrations or chaos make the near future seem hopeless, one can still look to the distant future for redemption. Hope is an eager state, closely aligned with approach motivation, and is negatively correlated with anxiety (McGregor et al., 2012b). Indeed, when people are confronted with anxiety-inducing threats, they respond by exaggerating hopeful commitment in alternative domains at near and far temporal distances as a way to activate RAM and suppress anxiety (reviewed in Jonas et al., 2014). But if all temporal horizons for hope, near and far, seem blocked, then people simmer in impotent anxiety, get depressed, and disengage from life. The combination of present life-dissatisfaction, salience of near-future failure, and mortality salience (that undermines distant future hope) made participants became depressed, demotivated, disinterested in personal goals, and less interested in living (Hayes et al., 2015).

Such across-the board withdrawal from life goes against the primal human motivation to strive, live, and love, however, (Ryan and Deci, 2000). Accordingly, such no-hope predicaments could be expected to vacillate between depression and fatalistic withdrawal on the one hand, and anxiety arising from frustrated efforts to re-engage with life on the other. Such vacillation would be conducive to ARR because the withdrawal would make people more willing to forsake normal goals and even die, but the anxiety would also orient them toward active participation in extreme ARR as a source of group-based, external agency to activate RAM for relief. ARR could be even more alluring because its utopian elements transcend the frustrating limits of the hopeless temporal world (Cohen et al., 2011).

It is important to note that neither threat nor hopelessness is synonymous with objective personal failure, disadvantage, or low SES. Hopelessness can refer to the plight of a group one identifies with (Wohl et al., 2010). It can also refer to a sense of futility about reaching whatever standard one has for oneself, high or low. Indeed, hopelessness may become particularly acute when one has ostensibly succeeded according to normative standards, but still feels dissatisfied. Being wealthy and vocationally successful, for example, but still feeling ostracized, socially excluded, alienated, or meaningless might make one feel especially hopeless and cynical about the viability of culturally available opportunities for a good life. Similarly, even in aﬄuent circumstances domestic dysfunction could trigger hopelessness about social goals.

### Affordance

#### Salient Narratives and Opportunities Conducive to Immediate, Concrete Engagement with Active Groups

The aforementioned combination of traits and threats should make RAM via concrete engagement in active groups particularly appealing. People intuitively turn to groups and consensus in anxious circumstances because doing so can help people either bolster or escape from their vulnerable or uncertain selves (e.g., Festinger et al., 1956; Solomon et al., 1991; Gardner et al., 2002; McGregor et al., 2005; Fritsche et al., 2008). Fusing one’s personal identity with a group identity can powerfully validate and bolster confidence in personal agency, liberating sacrifice and risky extremes for the sake of the group (Swann and Buhrmester, 2015). Groups can also support de-individuated identification as an escape from personal selfhood, and thereby promote enactment of group norms and beliefs even if they are anti-normative (Postmes and Spears, 1998). Authoritarian groups may further facilitate conformity and obedience beyond personal inclinations (Martin and Hewstone, 2003). Cleaving to group norms as a way to escape self-uncertainty is a theme long identified by existential philosophers and classic developmental psychological perspectives as described in the introduction. Authoritarian groups allow one to follow concrete orders, focusing on the concrete topics of what to do and how to do it instead of being responsible for grappling with uncertain moral quandaries about why. Authoritarian groups that explicitly specify immediate engagement in concrete action should be especially appealing for anxious and identity-weak people (Vallacher and Wegner, 1989; Baumeister, 1991; Hogg and Adelman, 2013; Tran et al., unpublished manuscript).

Indeed, people with barriers to personal agency react to threats by cleaving specifically to active and agentic groups, presumably because the groups’ agency can be internalized as a surrogate or splint for personal agency (Hogg and Adelman, 2013; Stollberg et al., 2015; cf. Landau et al., 2015). Consistent with this group-based agency view, short experimental manipulations of ingroup affirmation or outgroup derogation can activate approach motivated states (McGregor, unpublished data). Approach motivation is an eager, confident, and resilient state resistant to anxious distress (McGregor et al., 2005, 2012b; Drake and Myers, 2006; Nash et al., 2011).

Concrete group-identification allows anxious and identity-weak people to escape the self in two ways—by eliminating the ambiguities of moral abstraction by immersing in concrete and immediate experience, and by splinting their problematic personal identity with the authoritarian group identity. The preference for active engagement is consistent with the finding that previous involvement in active groups, like soccer teams, service groups, or religious groups is a predictor of engagement in ARR that is surprisingly superficial in idealism beyond the hostile and extremely black and white morality of us = good vs. them = bad (Atran, 2010). Physical action is also more concrete than ideas and values, and so would further appeal to the anxious and identity-weak. IS training manuals that advise recruits on the minutia of concrete planning practicalities like bringing knee and elbow pads in readiness for immediate action may thus feel exhilarating for anxious and identity-weak people. Concrete engagement, even in suicidal aggression could feel like a welcome adventure for oppositional, anxious, and identity-weak people, fused to or highly identified with the power of active groups (Baumeister, 1991; Hogg, 2014; Hayes et al., 2015; Swann and Buhrmester, 2015). This may explain the explicitly concrete declaration of Jihad against tyrants in the al-Qaeda terrorism manual that states (from Post, 2005):

“The confrontation that we are calling for with the apostate regimes does not know Socratic debates, Platonic ideals, nor Aristotelian diplomacy. But it knows the dialog of bullets, the ideals of assassination, bombing and destruction, and the diplomacy of the cannon and the machine gun.”

The concrete appeal of ARR may gel when narratives rationalizing participation in ARR become consensually endorsed in already-fused buddy groups (Atran, 2010) with members searching for greater purpose and meaning in life (Atran, 2015). As described next, we propose that ARR-compelling narratives involve conspiracy theory grievances, exaggerated cosmic significance of participating in resistance, rationalization for hostile retribution, and perceived legitimacy of unfalsifiable truth claims (Armstrong, 2000, 2014; Barrett, 2014a; Ali, 2015).

#### Conspiracy Narratives

Experimentally manipulated threats related to anxiety (mortality salience, low power, low control, frustration, and uncertainty) increase belief in superstitions and conspiracy theories (reviewed in Landau et al., 2015). The prevailing explanation for such experimentally induced threat-to-conspiracy effects is that belief in externally controlling phenomena like conspiracy plots or superstitious agencies helps make the world seem less random and more orderly. Compensatory control theorists see the need for order as so fundamental that people are even willing to accept the existence of (delusional) enemies and evil forces if it can reduce anxiety by restoring a perception of order. Even sinister order is preferable to chaos (Sullivan et al., 2010).

Another possible interpretation of such anxiety-induced superstitious/conspiracy effects is that anxiety-related threats cause a BIS-induced anxious vigilance that makes people over-notice strange and malevolent possibilities, and to experience the world as *more* out of control and random (as found by Agroskin and Jonas, 2013). If anything could happen, then maybe superstitions, evil forces, and conspiracies are true. Moreover, the same combination of external threats and identity-weak personality traits (e.g., low hope, low self-esteem, low efficacy, and low idealism) that result in persistent anxiety and depression (Hayes et al., 2015) also increase superstitious and supernatural belief (Prentice, unpublished master thesis; Prentice et al., unpublished data; Ferriday, unpublished master thesis). We are currently investigating whether this specific combination of personality traits and external threats will also predict belief in conspiracy theories, and if so whether this heightened belief is a palliative antidote to anxiety (following compensatory control theory), a symptom of anxiety (following the vigilant anxiety view), or both.

Whatever the mechanism, anxiety-related threats heighten paranoid belief in conspiracy theories and enemyship. Salience of consensually endorsed conspiracy theories should accordingly be an important risk factor for ARR. In the Islamist context, ubiquitous narratives about the hypocritical and callous abuses of the Great Satan West and/or Zionist or Shia forces rationalize Islamist ARR, and make it seem legitimate. Cultural endorsement reifies the conspiracies (e.g., Hardin and Higgins, 1996) and creates a ready focus for anxious vigilance aroused by hopeless circumstances, and a convenient scapegoat target to fight against with palliative zeal. Conspiracy narratives were similarly used by Christians to justify pogroms against Jews and Crusades against Muslims during historical periods where Christians were the ones experiencing cultural and existential threat (Durant, 1950; Armstrong, 2000, 2014). The prevalence and cultural acceptance of conspiracy theories should therefore be an affordance that promotes ARR.

#### Cosmic Moral Struggle

Once identified, the same combination of factors that makes conspiracy theories attractive can also create extreme moral conviction about polarized forces of good (us) vs. evil (them). An ARR perpetrator described his motivation for ARR as follows (from Sim, 2013):

“Jihad is a war purely to enforce Allah’s word…to protect Islam and Muslims…widen Islamic power… and spread kindness and the truth... It seemed that there was no effective way to effect change and to stop all the destruction except to wage jihad—a war between good and evil. When there is a war between good and evil, sinners will be afraid of the swords of the mujahidin. Thus, cruelty will be demolished gradually. My involvement with bombing has a personal purpose and hope. We bomb those whom we think of as the enemy and also in order to fulfill the obligation of jihad for Allah… On the battlefield, it will be seen who is good and who is evil … Jihad … is a pure war which is ordered by Allah. I always pray to be given the ability and opportunities to carry out my obligation to do jihad.”

As reviewed in Jonas et al. (2014) hundreds of experiments have demonstrated that anxiety-related threats make people go to moral extremes. The direction of the extremist urge under threat can be guided by salient cultural norms, whether antisocial or prosocial. Salient cultural or religious narratives that promote polarized views of goodness (us) vs. evil (them) and make every small action by an individual on behalf of the fight seem significant may thus provide especially attractive refuge (Pyszczynski et al., 2006; Kruglanski et al., 2014). Such oversimplified cosmic struggle narratives are like ultra-conspiracy theories with opportunity for action-oriented participation. Frustrations and conflicts on the ground become infused with universal significance, unencumbered by complicating details of concrete reality. Aggressive justice restoration becomes a simple and easily accessible ideology that the identity-weak can readily harbor in private imagination. For people seeking relief from temporal frustrations and uncertainties, exiting the temporal realm and concretely fighting for the sake of transcendent abstractions is intoxicating. Pure ideals and anger can activate sanguine, approach motivated states economically and anonymously in one’s own mind (McGregor, 2007; Carver and Harmon-Jones, 2009). These oversimplified idealistic surrogates may be especially attractive to identity-weak people who feel morally bereft. Indeed, idealistically bereft converts with little religious education often become among the most zealous perpetrators of ARR (Barrett, 2014a,b; Atran, 2015). For them, the utopian appeal of IS may be particularly alluring.

#### Violence Justification

Aggression is a primal reaction to frustration, especially if aggressive cues are salient (Berkowitz, 1989). This may be because anger is a powerful lever for approach-motivation (Carver and Harmon-Jones, 2009), and approach motivated states mute anxious distress (Corr, 2008; Nash et al., 2012). Imagining and enacting aggressive revenge should therefore feel rewarding in frustrating and anxious circumstances. Classic and new research on reactive aggression emphasizes that just as ambient cues moderate the direction of antisocial vs. prosocial ideology after threats, the ambient cues moderate the extent to which frustration causes aggression (Berkowitz, 1989; reviewed in Jonas et al., 2014). Salient narratives that prime and justify ARR are therefore essential for its reactive appeal. Enthusiasm for punitive justice may initially get provoked by outrage over ingroup suffering, and become amplified by conspiracy theories with cosmic significance that ignites angry eagerness to fight against the evil unfairness. Narratives that excuse ARR violence as holy, necessary, and just—‘they deserve it’ (e.g., as described in Sim, 2013) may be necessary to release this eager fury from normal social constraints against aggression. Indeed, group consensus can provide validation and legitimization for murderous activities that would otherwise be considered universally immoral and more stressful to participate in (Webber et al., 2013).

The same factors that make aggressive narratives appealing may also heighten willingness to subject oneself to risky commitments, reduce capacity for consideration of what one has to lose, and reduce capacity for awareness of others’ suffering. Threat-induced RAM makes people take more risks and become insensitive to distress. Eager approach cues predominate, and the salience of aversive stimuli fade to seem less motivationally relevant (Nash, unpublished doctoral dissertation). Eager religious narratives bent around the psychological reward for martyrdom, and courageous aggression against infidels with callous disregard for their suffering would therefore resonate with the motivational configuration of ARR. The recent spate of Islamist ARR shares a common narrative originating from Wahabi/Salafist thought that justifies extreme violence against apostates and infidels (Barrett, 2014b). Such callous and violence-encouraging narratives have gained traction in radical sects of all major religious traditions during periods of cultural anxiety and hopelessness (Armstrong, 2000, 2014; cf. Ali, 2015).

#### Unfalsifiability

Supernatural authority also helps preserve dubious distortions and rationalizations that can make ARR seem legitimate. Anxiogenic threats cause anxious and identity-weak people to more strongly endorse transcendent religious explanations for personal well-being and world events (McGregor et al., 2010b, Study 1; Ferriday, unpublished master thesis). An advantage of supernatural religious narratives rooted in transcendent and historical authority beyond physical proof is that they can be unfalsifiably asserted without the inconvenience of evidence and without the possibility of conflict. They can be bolstered merely by appeals to traditional authority and social consensus. Accordingly, experimentally manipulated threats cause people to spontaneously frame their moral arguments as more unfalsifiable, and randomly assigned unfalsifiability causes arguments to become more extreme and aggressive against dissenters (Friesen et al., 2015). Religious unfalsifiability may therefore be an important affordance that both consolidates zeal and spurs confident and militant action for ARR (cf. Ali, 2015).

### Hypothesis Testing

#### In the Laboratory

To test our multifactor hypothesis blocks of experiments could separately test the personality, threat, and affordance variables while holding the active ingredients from the other blocks constant. We would predict a three way interaction with highest endorsement of ARR among participants whose personalities are most oppositional, anxious, and identity-weak (with conducive threat and affordance circumstances primed for all). The best constellation of specific traits for each of the latent personality variables could be modeled with structural equation modeling. The ARR scenario participants would be asked to rate for the dependent variable would be plausibly tailored to the sample (as in Pyszczynski et al., 2006).

The threat block of experiments could experimentally manipulate externally imposed threat with mortality, relationship-distress, system-collapse, or injustice salience (vs. neutral) manipulations. Hopelessness could be manipulated by randomly assigning participants to write about a high effort commitment that did not pay off vs. one that did pay off (as in Hayes et al., 2015) or about an ongoing ingroup injustice with no hope vs. hope for abatement. Participants preselected with conducive personalities and primed with the affordance variables should show highest endorsement of ARR in the external threat/hopeless circumstances condition.

To test the affordance block of variables, participants preselected for conducive personality traits and primed with the conducive threat variables would rate the appeal of six different randomly assigned versions of an ARR scenario. In five of the conditions only four of the affordance variables would be woven into the scenario. In the sixth condition, all five affordance variables would be present. ARR should be most prevalent in a sixth condition with all five affordance variables present.

Once each factor was established, a final study could test the entire model in a large 2 (personality) × 2 (threat) × 2 (affordance) between subjects design. Participants would be preselected based on combined high vs. low personality predisposition and the threat and affordance factors would be experimentally manipulated. Highest endorsement of ARR would be expected in the conducive personality/threat/affordance cell.

#### Interview Studies

Biographical analysis should also reveal personality × threat × affordance factors among ARR perpetrators. Thousands of foreign fighters are returning home from participation in ARR, many who are disillusioned and might be willing to participate in research designed to help understand the phenomenon. They could be comprehensively tested and interviewed to assess the adequacy of the proposed multifactor hypotheses if researchers could get access to them (Sim, 2013; Sageman, 2014). Interviews could also be conducted in international hotbeds that produce inordinately high numbers of ARR recruits to test for prevalence of conducive factors (The Soufan Group, 2015).

#### Real World

Interventions aimed at mitigating personality, threat, or affordance factors could be targeted or observed while controlling for the other two factors. Many aspects of personality trait dispositions are stable across time, but two aspects of ARR conducive personality might be amenable to intervention. Traits rooted in anxiety-related insecurities are affected by relationship responsiveness and security (e.g., Bokhorst et al., 2003; DeHart et al., 2006; Fearon et al., 2006). Policy or programs that provided access to affordable daycare or other resources to reduce stress for caregivers could help. Public education about importance of responsive connection and autonomy support for children and ill-effects of authoritarian parenting could also be promoted (Ryan and Deci, 2000).

A second aspect of ARR conducive personality amenable to intervention is identity-weakness. Provision of adequate structure to support youth’s developing sense of efficacy and autonomy, and interventions that promote pro-social values and belonging in experientially engaging groups could help bolster authentic intrinsic motivation to stabilize identity and inoculate against ARR (Ryan and Deci, 2000; Walton and Cohen, 2007, 2011; cf. Cohen and Sherman, 2014). The same manipulations could also decrease anxious reactivity (Creswell et al., 2006).

A set of quasi-experimental and intervention studies could also focus on factors affecting threat variables. They could assess whether ARR varies with externally threatening and hopeless social conditions, e.g., exposure to economic disruption, sanctions, invasions, occupations, mass-displacements, wars, political instability, institutional corruption, natural disasters, racial profiling, police brutality, judicial unfairness, unemployment, social ostracism vs. integration, accessibility of social services and acculturation support. Intervention experiments could follow up by enriching one of two matched communities with social programs that build infrastructure and systems to help culturally vulnerable immigrants cope, feel welcome, and able to hope. ARR incidence could be compared before and after interventions as well as to incidence in matched communities not receiving the interventions.

Similar studies could assess prevalence of the affordance factors and ARR. Countering ARR narratives with accessible and credible alternatives has been identified as a crucial intervention point (The Soufan Group, 2013; Atran, 2015). Effects of providing opportunity for active engagement in meaningful group activities suffused by prosocial narratives could be tested. Non-radical religious engagement might be particularly effective. Religious devotion is a powerful and mutable human response to the pervasive appeal of consensual moral clarity in action. Anxious humans yearn for moral devotion. The best defense against narratives that support the darker mutations might be opportunity for participation in narratives that uphold the lighter ones, and intrinsic religiosity is universally prosocial (Jonas and Fischer, 2006; Armstrong, 2009; Schumann et al., 2014; Shariff et al., 2015). To be effective, the counter-narratives would need to equally address local grievances, and have as much opportunity for experiential linkage to active engagement, adventure, and intimate group involvement as ARR narratives do (The Soufan Group, 2013; Atran, 2015).

Intervention studies in countries where most young people go to school could test the effects of counter-ARR education. Comparative religion curricula could reveal the history of world religions and weakness of violent religious arguments. Objectively debunked conspiracy theories, such as The Protocols of the Elders of Zion, could also be exposed, and examples of inter-religious cooperation could be featured (political apologies for actual historical injustices could also help; Blatz et al., 2009). The history and psychology of extreme, rigid belief systems could also be taught, summarizing the now 100s of experiments showing that intuitive moral certainty can be defensive and is an unreliable index of objective truth. Developmental and historical aberrations toward zealous extremism and aggression could be revealed as such and the centrality of mystery, humility, and compassion as fundamental to religious piety across traditions could be taught (Armstrong, 2000, 2009).

Importantly, effects of these real-world interventions would need to be carefully evaluated. De-radicalization and risk-reduction programs that try to mitigate threat and affordance factors for at-risk individuals are already in place in the United Kingdom, Indonesia, Singapore, Sweden, Netherlands, Spain, Denmark, and elsewhere. They already combine elements of integration and support for immigrants, community resilience resources, and religious education with tools to counter ARR propaganda. What is missing is systematic program evaluation of what works and what does not work (Qatar International Academy for Security Studies, 2010). We submit that our hypotheses, derived from psychological theory and research, could help guide interventions and program evaluations in the real world. Given their current uncertainty about root causes of ARR, it is not surprising that politicians are reluctant to wade into that theoretical murkiness. Strong-arm punishment is a safer strategy. The Conservative Prime Minister of Canada recently dismissed interest in root causes of ARR with the unapologetic quip “this is not a time to commit sociology” after the arrest of religious radicals who had plotted to blow up a Canadian train (Fitzpatrick, 2013). He then announced a new bombing campaign against IS, and pushed through new anti-terror laws with unprecedented violation of civil rights.

Our hypotheses predict that punitive and fairness-violating social policies are as likely to exacerbate as quell ARR. ARR groups are fueled by an “ideology of protest” rooted in local grievances about maltreatment (The Soufan Group, 2013; The Economist, 2015). Brutal crackdowns fuel this fire. Post (2005) concluded that religious violence is driven by the psychological appeal of displaced aggression, for people who feel oppressed and hopeless. He further emphasized how cultural narratives across generations breed deep ideological hatred. His view converges with ours, in its conclusion that the appeal of ARR is psychological, and should be countered with psychological, at least as much as military intelligence. Atran (2015) similarly identified a psychological motivation for ARR as primarily idealistic:

Most have had no traditional religious education, and are often “born again” into a socially tight, ideologically narrow but world-spanning sense of religious mission... Violent extremism represents not the resurgence of traditional cultures, but their collapse, as young people unmoored from millennial traditions flail about in search of a social identity that gives personal significance and glory.

He further notes the importance of action groups that provide opportunity to act with “deep conviction of moral virtue,” and that “sacred value must be fought with other sacred values.” “When, as now, the focus is on military solutions and police interdiction, matters have already gone way too far. If that focus remains, we lose the coming generation.” We hope that the recent empirical advances in personality and social psychological knowledge about root causes of ARR together with new knowledge derived from the hypotheses described above will augment converging insights from other disciplines, and help make wisdom drawn from psychological science a credible option for leaders grappling with ARR.

## Conflict of Interest Statement

The authors declare that the research was conducted in the absence of any commercial or financial relationships that could be construed as a potential conflict of interest.

## References

[B1] Adorno T. W. Frenkel-Brunswik E. Levinson D. J. Sanford R. N. (1950). *The Authoritarian Personality.* New York, NY: Harper.

[B2] Agroskin D. Jonas E. (2013). Controlling death by defending ingroups: mediational insights into terror management and control restoration. *J. Exp. Soc. Psychol.* 49 1144–1158. 10.1016/j.jesp.2013.05.014

[B3] Ali A. H. (2015). *Heretic: Why Islam needs a Reformation Now.* New York, NY: Harper Collins.

[B4] Allport G. W. (1943). The ego in contemporary psychology. *Psychol. Rev.* 50 451–478. 10.1037/h0055375

[B5] Allport G. W. Ross J. M. (1967). Personal religious orientation and prejudice. *J. Pers. Soc. Psychol.* 5 432–443. 10.1037/h0021212 6051769

[B6] Anderson C. A. Dill K. E. (2000). Video games and aggressive thoughts, feelings, and behavior in the laboratory and in life. *J. Pers. Soc. Psychol.* 78 772–790. 10.1037/0022-3514.78.4.772 10794380

[B7] Ansbacher H. L. Ansbacher R. R. (1956). *The Individual Psychology of Alfred Adler.* New York, NY: Basic Books.

[B8] Armstrong K. (2000). *The Battle for God: A History of Fundamentalism.* New York, NY: Ballantine Books.

[B9] Armstrong K. (2006). *The Great Transformation: The Beginnings of our Religious Traditions.* New York, NY: Alfred A. Knopf.

[B10] Armstrong K. (2009). *The Case for God. What Religion Really Means.* New York, NY: Alfred A. Knopf.

[B11] Armstrong K. (2014). *Fields of Blood: Religion and the History of Violence.* New York, NY: Random House.

[B12] Atran S. (2010). *Talking to the Enemy: Faith, Brotherhood, and the (un) Making of Terrorists.* New York, NY: Harper Collins.

[B13] Atran S. (2015). *The Role of Youth in Countering Violent Extremism and Promoting Peace. Address to the UN Security Council, Ministerial Debate.* Available at: http://blogs.plos.org/neuroanthropology/2015/04/25/scott-atran-on-youth-violent-extremism-and-promoting-peace/ [accessed April, 23 2015].

[B14] Baker L. A. Raine A. Liu J. Jacobson K. C. (2008). Differential genetic and environmental influences on reactive and proactive aggression in children. *J. Abnorm. Child Psychol.* 36 1265–1278. 10.1007/s10802-008-9249-1 18615267 PMC2609906

[B15] Barnes H. E. (1973). *Sartre.* Philadelphia: Lippincott.

[B16] Barrett R. (2014a). *Foreign Fighters in Syria. The Soufan Group.* Available at: http://soufangroup.com/foreign-fighters-in-syria/ [accessed June 2 2014].

[B17] Barrett R. (2014b). *The Islamic State. The Soufan Group.* Available at: http://soufangroup.com/the-islamic-state/ [accessed October 28 2014].

[B18] Barrett R. (2015). ISIS will fall through internal revolt. *The New York Times* 2015 Available at: http://www.nytimes.com/roomfordebate/2015/06/01/can-victory-over-isis-be-more-than-a-mirage/isis-will-fall-through-internal-revolt [accessed June 1 2014].

[B19] Baumeister R. F. (1986). *Identity: Cultural Change and the Struggle for Self.* New York: Oxford University Press.

[B20] Baumeister R. F. (1990). Suicide as escape from self. *Psychol. Rev.* 97 90–113. 10.1037/0033-295X.97.1.90 2408091

[B21] Baumeister R. F. (1991). *Escaping the Self.* New York, NY: Basic Books.

[B22] BBC News. (2014). *Islamic State: Can Its Savagery Be Explained?* Available at: http://www.bbc.com/news/world-middle-east-29123528 [accessed September 9 2014].

[B23] BBC News. (2015). *‘Jihadi John’ Named as Mohammed Emwazi from London.* Available at: http://www.bbc.com/news/uk-31637090 [accessed February 26 2015].

[B24] Beaver K. M. Schutt J. E. Boutwell B. B. Ratchford M. Roberts K. Barnes J. C. (2009). Genetic and environmental influences on levels of self-control and delinquent peer affiliation results from a longitudinal sample of adolescent twins. *Crim. Justice Behav.* 36 41–60. 10.1177/0093854808326992

[B25] Berkowitz L. (1989). Frustration-aggression hypothesis: examination and reformulation. *Psychol. Bull.* 106 59 10.1037/0033-2909.106.1.59 2667009

[B26] Black C. L. Goldstein K. E. LaBelle D. R. Brown C. W. Harmon-Jones E. Abramson L. Y. (2014). Behavioral approach system sensitivity and risk taking interact to predict left-frontal EEG asymmetry. *Behav. Ther.* 45 640–650. 10.1016/j.beth.2014.01.006 25022775 PMC4100074

[B27] Blatz C. W. Schumann K. Ross M. (2009). Government apologies for historical injustices. *Polit. Psychol.* 30 219–241. 10.1111/j.1467-9221.2008.00689.x

[B28] Bokhorst C. L. Bakermans-Kranenburg M. J. Fearon R. M. P. vanIjzendoorn M. H. Fonagy P. Schuengel C. (2003). The importance of shared environment in mother-infant attachment security: a behavioral genetic study. *Child Dev.* 74 1769–1782. 10.1046/j.1467-8624.2003.00637.x 14669895

[B29] Burke B. L. Martens A. Faucher E. H. (2010). Two decades of terror management theory: a meta-analysis of mortality salience research. *Pers. Soc. Psychol. Rev.* 14 155–195. 10.1177/1088868309352321 20097885

[B30] Bushman B. J. (1995). Moderating role of trait aggressiveness in teh effects of violent media on aggression. *J. Pers. Soc. Psychol.* 69 950–960. 10.1037/0022-3514.69.5.950 7473040

[B31] Bushman B. J. Baumeister R. F. (1998). Threatened egotism, narcissism, self-esteem, and direct and displaced aggression: does self-love or self-hate lead to violence. *J. Pers. Soc. Psychol.* 75 219–229. 10.1037/0022-3514.75.1.219 9686460

[B32] Campbell W. K. Rudich E. A. Sedikides C. (2002). Narcissism, self-esteem, and the positivity of self-views: two portraits of self-love. *Pers. Soc. Psychol. Bull.* 28 358–368. 10.1177/0146167202286007

[B33] Carver C. S. Harmon-Jones E. (2009). Anger is an approach-related affect: evidence and implications. *Psychol. Bull.* 135 183–204. 10.1037/a0013965 19254075

[B34] Carver C. S. Scheier M. F. (1998). *On the Self-Regulation of Behavior.* New York, NY: Cambridge University Press 10.1017/CBO9781139174794

[B35] Chang L. Arkin R. M. (2002). Materialism as an attempt to cope with uncertainty. *Psychol. Mark.* 19 389–406. 10.1002/mar.10016

[B36] Cohen F. Sullivan D. Solomon S. Greenberg J. Ogilvie D. M. (2011). Finding everland: flight fantasies and the desire to transcend mortality. *J. Exp. Soc. Psychol.* 47 88–102. 10.1016/j.jesp.2010.08.013

[B37] Cohen G. L. Sherman D. K. (2014). The psychology of change: self-affirmation and social psychological intervention. *Annu. Rev. Psychol.* 65 333–371. 10.1146/annurev-psych-010213-115137 24405362

[B38] Corr P. J. (2008). *The Reinforcement Sensitivity Theory of Personality.* Cambridge: Cambridge University Press 10.1017/CBO9780511819384

[B39] Costa P. T. McCrae R. R. (1991). Facet scales for agreeableness and conscientiousness: a revision of the NEO personality inventory. *Pers. Individ. Dif.* 12 887–898. 10.1016/0191-8869(91)90177-D

[B40] Creswell J. D. Welch W. T. Taylor S. E. Sherman D. K. Gruenewald T. L. Mann T. (2006). Affirmation of personal values buffers neuroendocrine and psychological stress responses. *Psychol. Sci.* 16 846–851. 10.1111/j.1467-9280.2005.01624.x 16262767

[B41] Crocker J. Niiya Y. Mischkowski D. (2008). Why does writing about important values reduce defensiveness? Self-affirmation and the role of positive other-directed feelings. *Psychol. Sci.* 19 740–747. 10.1111/j.1467-9280.2008.02150.x 18727791

[B42] Deci E. L. Ryan R. M. (1991). “A motivational approach to self: integration in personality,” in *Nebraska Symposium on Motivation: Perspectives on Motivation* Vol. 38 ed. Dienstbier R. (Lincoln: University of Nebraska Press), 237–288. 2130258

[B43] DeHart T. Pelham B. W. Tennen H. (2006). What lies beneath: parenting style and implicit self-esteem. *J. Exp. Soc. Psychol.* 42 1–17. 10.1016/j.jesp.2004.12.005

[B44] Dodgson P. G. Wood J. V. (1998). Self-esteem and the cognitive accessibility of strengths and weaknesses after failure. *J. Pers. Soc. Psychol.* 75 178–197. 10.1037/0022-3514.75.1.178 9686458

[B45] Dollard J. Doob L. W. Miller N. E. Mowrer O. H. Sears R. R. (1939). *Frustration and Aggression.* New Haven, CT: Yale University Press 10.1037/10022-000

[B46] Drake R. A. Myers L. R. (2006). Visual attention, emotion, and action tendency: feeling active or passive. *Cogn. Emot.* 20 608–622. 10.1080/02699930500368105

[B47] Durant W. (1950). *The Age of Faith.* New York, NY: Simon and Schuster.

[B48] Durkheim E. (1897/1951). *Suicide: A Study in Sociology.* Trans. Spaulding J. A. Simpson G. Glencoe, IL: Free Press.

[B49] Durkheim E. (1976/1912). *The Elementary forms of the Religious Life.* London: Routledge.

[B50] Elliot A. J. McGregor H. A. (1999). Test anxiety and the hierarchical model of approach and avoidance achievement motivation. *J. Pers. Soc. Psychol.* 76 628–644. 10.1037/0022-3514.76.4.628 10234849

[B51] Erikson E. H. (1959). *Identity and the Life Cycle: Selected Papers.* New York, NY: International Universities Press.

[B52] Fearon R. M. P. Van IJzendoorn M. H. Fonagy P. Bakermans-Kranenburg M. J. Schuengel C. Bokhorst C. L. (2006). In search of shared and nonshared environmental factors in security of attachment: a behavior-genetic study of the association between sensitivity and attachment security. *Dev. Psychol.* 42 1026–1040. 10.1037/0012-1649.42.6.1026 17087539

[B53] Festinger L. (1950). Informal social communication. *Psychol. Rev.* 57 271–282. 10.1037/h0056932 14776174

[B54] Festinger L. (1957). *A Theory of Cognitive Dissonance.* Evanston, IL: Row, Peterson and Company.

[B55] Festinger L. Riecken H. W. Schachter S. (1956). *When Prophecy Fails.* Minneapolis, MN: University of Minnesota Press 10.1037/10030-000

[B56] Fitzpatrick M. (2013). Harper on terror arrests: not a time to commit ‘Sociology.’. *CBC News* Available at: http://www.cbc.ca/news/politics/harper-on-terror-arrests-not-a-time-for-sociology-1.1413502 [accessed April 25 2013].

[B57] Freud A. (1946). *The Ego and the Mechanisms of Defence.* New York, NY: International Universities Press.

[B58] Friesen J. P. Campbell T. H. Kay A. C. (2015). The psychological advantage of unfalsifiability: the appeal of untestable religious and political ideologies. *J. Pers. Soc. Psychol.* 108 515 10.1037/pspp0000018 25402678

[B59] Fritsche I. Jonas E. Ablasser C. Beyer M. Kuban J. Manger A. M. (2013). The power of we: evidence for group-based control. *J. Exp. Soc. Psychol.* 49 19–32. 10.1016/j.jesp.2012.07.014

[B60] Fritsche I. Jonas E. Fankhänel T. (2008). The role of control motivation in mortality salience effects on ingroup support and defense. *J. Pers. Soc. Psychol.* 95 524–541. 10.1037/a0012666 18729692

[B61] Fritsche I. Jonas E. Kessler T. (2011). Collective reactions to threat: implications for intergroup conflict and for solving societal crises. *Soc. Issues Policy Rev.* 5 101–136. 10.1111/j.1751-2409.2011.01027.x

[B62] Fromm E. (1941). *Escape from Freedom.* New York, NY: Farrar & Rinehart, Inc.

[B63] Fromm E. (1947). *Man for Himself: An Inquiry into the Psychology of Ethics.* New York: Holt, Rinehart, and Winston.

[B64] Fromm E. (1973). *The Anatomy of Human Destructiveness.* Greenwich, CT: Fawcett Publications.

[B65] Gardner W. L. Gabriel S. Hochschild L. (2002). When you and I are “we,” you are not threatening: the role of self-expansion in social comparisons. *J. Pers. Soc. Psychol.* 82 239–251. 10.1037/0022-3514.82.2.239 11831413

[B66] Gay P. (1989). *The Freud Reader.* New York, NY: Norton.

[B67] Geertz C. Banton M. (1966). “Religion as a cultural system,” in *Anthropological Approaches to the Study of Religion*, ed. Banton M. (London: Tavistock), 1–46.

[B68] Ginges J. Hansen I. Norenzayan A. (2009). Religion and support for suicide attacks. *Psychol. Sci.* 20 224–230. 10.1111/j.1467-9280.2009.02270.x 19170938

[B69] Graham J. Haidt J. Nosek B. A. (2009). Liberals and conservatives rely on different sets of moral foundations. *J. Pers. Soc. Psychol.* 96 1029–1046. 10.1037/a0015141 19379034

[B70] Gray J. A. McNaughton N. (2000). *The Neuropsychology of Anxiety: An Enquiry into the Functions of the Septo-Hippocampal System.* New York, NY: Oxford Press.

[B71] Greenaway K. H. Storrs K. R. Philipp M. C. Louis W. R. Hornsey M. J. Vohs K. D. (2015). Loss of control stimulates approach motivation. *J. Exp. Soc. Psychol.* 56 235–241. 10.1016/j.jesp.2014.10.009

[B72] Hardin C. D. Higgins E. T. (1996). “Shared reality: how social verification makes the subjective objective,” in *Handbook of Motivation and Cognition: The Interpersonal Context* Vol. 3 eds Sorrentino R. M. Higgins E. T. (New York, NY: Guilford), 28–84.

[B73] Harmon-Jones E. Amodio D. M. Harmon-Jones C. (2009). Action-based model of dissonance: a review, integration, and expansion of conceptions of cognitive conflict. *Adv. Exp. Soc. Psychol.* 41 119–166.

[B74] Harmon-Jones E. Gable P. A. Price T. F. (2012). The influence of affective states varying in motivational intensity on cognitive scope. *Front. Integr. Neurosci.* 6:73 10.3389/fnint.2012.00073 PMC3437552 22973207

[B75] Harmon-Jones E. Harmon-Jones C. Price T. F. (2013). What is approach motivation? *Emot. Rev.* 5 291–295. 10.1177/1754073913477509

[B76] Harmon-Jones E. Harmon-Jones C. Serra R. Gable P. A. (2011). The effect of commitment on relative left frontal cortical activity: tests of the action-based model of dissonance. *Pers. Soc. Psychol. Bull.* 37 395–408. 10.1177/0146167210397059 21307178

[B77] Hayes J. Ward C. McGregor I. (2015). Why bother? Death, failure, and fatalistic withdrawal from life. *J. Pers. Soc. Psychol*. 10.1037/pspp0000039 [Epub ahead of print]. 25915129

[B78] Heine S. J. Proulx T. Vohs K. D. (2006). The meaning maintenance model: on the coherence of social motivations. *Pers. Soc. Psychol. Rev.* 10 88–110. 10.1207/s15327957pspr1002_1 16768649

[B79] Higgins A. (2015). A Norway town and its pipeline to Jihad in Syria. *The New York Times* Available at: http://www.nytimes.com/2015/04/05/world/europe/a-norway-townand-its-pipeline-to-jihad-in-syria.html?_r=0 [accessed June 4 2015].

[B80] Higgins E. T. (1996). The “Self Digest”: self-knowledge serving self-regulatory functions. *J. Pers. Soc. Psychol.* 71 1062–1083. 10.1037/0022-3514.71.6.1062 8979379

[B81] Hirschberger G. Pyszczynski T. Ein-Dor T. (2009). Vulnerability and vigilance: threat awareness and perceived adversary intent moderate the impact of mortality salience on intergroup violence. *Pers. Soc. Psychol. Bull.* 35 597–607. 10.1177/0146167208331093 19208903

[B82] Hirsh J. B. Inzlicht M. (2008). The devil you know neuroticism predicts neural response to uncertainty. *Psychol. Sci.* 19 962–967. 10.1111/j.1467-9280.2008.02183.x 19000202

[B83] Hirsh J. B. Kang S. K. (2015). Mechanisms of identity conflict uncertainty, anxiety, and the behavioral inhibition system. *Pers. Soc. Psychol. Rev.* 10.1177/1088868315589475 [Epub ahead of print]. 26048875

[B84] Hogeveen J. Inzlicht M. Obhi S. S. (2014). Power changes how the brain responds to others. *J. Exp. Psychol. Gen.* 143 755–762. 10.1037/a0033477 23815455

[B85] Hogg M. A. (2014). From uncertainty to extremism: social categorization and identity processes. *Curr. Dir. Psychol. Sci.* 23 338–342. 10.1177/0963721414540168

[B86] Hogg M. A. Adelman J. (2013). Uncertainty-identity theory: extreme groups, radical behavior, and authoritarian leadership. *J. Soc. Issues* 69 436–454. 10.1111/josi.12023

[B87] Horney K. (1950). *Neurosis and Human Growth.* New York, NY: Norton.

[B88] Jakobwitz S. Egan V. (2006). The ’dark triad’ and normal personality traits. *Pers. Individ. Dif.* 40 331–339. 10.1016/j.paid.2005.07.006

[B89] James W. (1902/1958).*The Varieties of Religious Experience.* New York, NY: Mentor.

[B90] Jonas E. Fischer P. (2006). Terror management and religion: evidence that intrinsic religiousness mitigates worldview defense following mortality salience. *J. Pers. Soc. Psychol.* 91 553–567. 10.1037/0022-3514.91.3.553 16938037

[B91] Jonas E. McGregor I. Klack L. Agroskin J. Fritsche D. Holbrook I. (2014). “Threat and defense: from anxiety to approach,” in *Advances in Experimental Social Psychology* Vol. 49 eds Olson J. M. Zanna M. P. (San Diego, CA: Academic Press), 219–286. 10.1016/b978-0-12-800052-6.00004-4

[B92] Jones D. N. Paulhus D. L. (2010). “Differentiating the dark triad within the interpersonal circumplex,” in *Handbook of Interpersonal Theory and Research*, eds Horowitz L. M. Strack S. N. (New York, NY: Guilford), 249–267.

[B93] Jordan C. H. Spencer S. J. Zanna M. P. (2005). Types of high self-esteem and prejudice: how implicit self-esteem relates to ethnic discrimination among high explicit self-esteem individuals. *Pers. Soc. Psychol. Bull.* 31 693–702. 10.1177/0146167204271580 15802663

[B94] Jost J. T. Glaser J. Kruglanski A. W. Sulloway F. J. (2003). Political conservatism as motivated social cognition. *Psychol. Bull.* 129 339–375. 10.1037/0033-2909.129.3.339 12784934

[B95] Juhl J. Routledge C. (2010). Structured terror: further exploring the effects of mortality salience and personal need for structure on worldview defense. *J. Pers.* 78 969–990. 10.1111/j.1467-6494.2010.00639.x 20573132

[B96] Kay A. C. Gaucher D. McGregor I. Nash K. (2010). Religious belief as compensatory control. *Pers. Soc. Psychol. Rev.* 14 37–48. 10.1177/1088868309353750 20040614

[B97] Kay A. C. Gaucher D. Napier J. Callan M. J. Laurin K. (2008). God and the government: testing a compensatory control explanation for the support of external systems of control. *J. Pers. Soc. Psychol.* 95 18–35. 10.1037/0022-3514.95.1.18 18605849

[B98] Keltner D. Gruenfeld D. H. Anderson C. (2003). Power, approach, and inhibition. *Psychol. Rev.* 110 265 10.1037/0033-295x.110.2.265 12747524

[B99] Kernberg O. (1975). *Borderline Conditions and Pathological Narcissism.* New York, NY: Jason Aronson.

[B100] Khan I. (2014). Horror paralyzes Pakistan after a methodical slaughter. *The New York Times* Available at: http://www.nytimes.com/2014/12/18/world/asia/horrorparalyzes-pakistan-after-a-methodical-slaughter.html?ref=asia&_r=3 [accessed December 17 2014].

[B101] Kohut H. (1971). *The Analysis of the Self.* New York, NY: International Universities Press.

[B102] Krizan Z. Johar O. (2015). Narcissistic rage revisited. *J. Pers. Soc. Psychol.* 108 784–801. 10.1037/pspp0000013 25545840

[B103] Kroger J. Marcia J. E. (2011). “The identity statuses: origins, meanings, and interpretations,” in *Handbook of Identity Theory and Research*, eds Schwartz S. J. Luyetx K. Vignoles V. L. (New York: Springer), 31–53.

[B104] Kruglanski A. W. Gelfand M. J. Bélanger J. J. Sheveland A. Hetiarachchi M. Gunaratna R. (2014). The psychology of radicalization and deradicalization: how significance quest impacts violent extremism. *Polit. Psychol.* 35 69–93. 10.1111/pops.12163

[B105] Kruglanski A. W. Shah J. Y. Pierro A. Mannetti L. (2002). When similarity breeds content: need for closure and the allure of homogeneous and self-resembling groups. *J. Pers. Soc. Psychol.* 83 648–662. 10.1037/0022-3514.83.3.648 12219860

[B106] La Guardia J. G. (2009). Developing who I am: a self-determination theory approach to the establishment of healthy identities. *Educ. Psychol.* 44 90–104. 10.1080/00461520902832350

[B107] Landau M. J. Kay A. C. Whitson J. A. (2015). Compensatory control and the appeal of a structured world. *Psychol. Bull.* 141 694–722. 10.1037/a0038703 25688696

[B108] Laurin K. Schumann K. Holmes J. G. (2014). A relationship with God? Connecting with the divine to assuage fears of interpersonal rejection. *Soc. Psychol. Pers. Sci.* 5 777–785. 10.1177/1948550614531800

[B109] Lewin K. (1933). “Environmental forces,” in *A Handbook of Child Psychology*, ed. Murchison C. (Worcester: Clark University Press), 590–625.

[B110] Lewin K. (1935). *A Dynamic Theory of Personality.* trans. Adams D. K. Zaner K. E. New York, NY: McGraw-Hill.

[B111] Lydon J. E. Zanna M. P. (1990). Commitment in the face of adversity: a value-affirmation approach. *J. Pers. Soc. Psychol.* 58 1040–1047. 10.1037/0022-3514.58.6.1040

[B112] Marcia J. (1980). “Identity in adolescence,” in *Handbook of Adolescent Psychology*, ed. Adelson J. (New York: Wiley).

[B113] Martin R. Hewstone M. (2003). *Social-Influence Processes of Control and Change: Conformity, Obedience to Authority and Innovation.* London: Sage.

[B114] McGregor I. (2003). “Defensive zeal: compensatory conviction about attitudes, values, goals, groups, and self-definition in the face of personal uncertainty,” in *Motivated Social Perception: The Ontario Symposium* Vol. 9 eds Spencer S. Fein S. Zanna M. (Mahwah, NJ: Erlbaum), 73–92.

[B115] McGregor I. (2004). “Zeal, identity, and meaning: Going to extremes to be one self,” in *Handbook of Experimental Existential Psychology*, eds Greenberg J. Koole S. L. Pyszczynski T. (New York, NY: Guilford), 182–199.

[B116] McGregor I. (2006a). Zeal appeal: the allure of moral extremes. *Basic Appl. Soc. Psychol.* 28 343–348. 10.1207/s15324834basp2804_7

[B117] McGregor I. (2006b). Offensive defensiveness: toward an integrative neuroscience of compensatory zeal after mortality salience, personal uncertainty, and other poignant self-threats. *Psychol. Inq.* 17 299–308. 10.1080/10478400701366977

[B118] McGregor I. (2007). “Personal projects as compensatory convictions: passionate pursuit and the fugitive self,” in *Personal Project Pursuit: Goals, Action and Human Flourishing*, eds Little B. R. Salmela-Aro K. Phillips S. D. (Mahwah, NJ: Lawrence Erlbaum Associates), 171–195.

[B119] McGregor I. Gailliot M. T. Vasquez N. A. Nash K. A. (2007). Ideological and personal zeal reactions to threat among people with high self-esteem: motivated promotion focus. *Pers. Soc. Psychol. Bull.* 33 1587–1599. 10.1177/0146167207306280 17933744

[B120] McGregor I. Haji R. Nash K. A. Teper R. (2008). Religious zeal and the uncertain self. *Basic Appl. Soc. Psychol.* 30 183–188. 10.1080/01973530802209251

[B121] McGregor I. Jordan C. H. (2007). The mask of zeal: low implicit self-esteem, and defensive extremism after self-threat. *Self Identity* 6 223–237. 10.1080/15298860601115351

[B122] McGregor I. Marigold D. C. (2003). Defensive zeal and the uncertain self: what makes you so sure? *J. Pers. Soc. Psychol.* 85 838–852. 10.1037/0022-3514.85.5.838 14599248

[B123] McGregor I. Nail P. R. Kocalar D. Haji R. (2013a). I’m OK, I’m OK: praise makes narcissists with low implicit self-esteem indifferent to the suffering of others. *Pers. Individ. Differ.* 55 655–659. 10.1016/j.paid.2013.05.007

[B124] McGregor I. Prentice M. Nash K. (2013b). Anxious uncertainty and reactive approach motivation (RAM) for religious, idealistic, and lifestyle extremes. *J. Soc. Issues* 69 537–563. 10.1111/josi.12028

[B125] McGregor I. Nail P. R. Marigold D. C. Kang S. (2005). Defensive pride and consensus: strength in imaginary numbers. *J. Pers. Soc. Psychol.* 89 978–996. 10.1037/0022-3514.89.6.978 16393029

[B126] McGregor I. Nash K. Prentice M. (2012a). “Religious zeal after goal frustration,” in *Extremism and the Psychology of Uncertainty*, eds Hogg M. A. Blaylock D. L. (Hoboken, NJ: Wiley-Blackwell), 147–164.

[B127] McGregor I. Prentice M. Nash K. (2012b). Approaching relief: compensatory ideals relieve threat-induced anxiety by promoting approach-motivated states. *Soc. Cogn.* 30 689–714. 10.1521/soco.2012.30.6.689

[B128] McGregor I. Nash K. A. Inzlicht M. (2009a). Threat, high self-esteem, and reactive approach motivation: electroencephalographic evidence. *J. Exp. Soc. Psychol.* 45 1003–1007. 10.1016/j.jesp.2009.04.011

[B129] McGregor I. Prentice M. Nash K. A. (2009b). Personal uncertainty management by reactive approach motivation. *Psychol. Inq.* 20 225–229. 10.1080/10478400903333460

[B130] McGregor I. Nash K. A. Mann N. Phills C. E. (2010a). Anxious uncertainty and reactive approach motivation (RAM). *J. Pers. Soc. Psychol.* 99 133–147. 10.1037/a0019701 20565191

[B131] McGregor I. Nash K. A. Prentice M. (2010b). Reactive approach motivation (RAM) for religion. *J. Pers. Soc. Psychol.* 99 148–161. 10.1037/a0019702 20565192

[B132] McGregor I. Newby-Clark I. R. Zanna M. P. (1999). “Remembering dissonance: simultaneous accessibility of inconsistent cognitive elements moderates epistemic discomfort,” in *Cognitive Dissonance: Progress on a Pivotal Theory in Social Psychology*, eds Harmon-Jones E. Mills J. (Washington, DC: American Psychological Association), 325–353. 10.1037/10318-013

[B133] McGregor I. Zanna M. P. Holmes J. G. Spencer S. J. (2001). Compensatory conviction in the face of personal uncertainty: going to extremes and being oneself. *J. Pers. Soc. Psychol.* 80 472–488. 10.1037/0022-3514.80.3.472 11300580

[B134] Mikulincer M. Florian V. (2000). Exploring individual differences in reactions to mortality salience: does attachment style regulate terror management mechanisms? *J. Pers. Soc. Psychol.* 79 260–273. 10.1037/0022-3514.79.2.260 10948979

[B135] Miles D. R. Carey G. (1997). Genetic and environmental architecture on human aggression. *J. Pers. Soc. Psychol.* 72 207–217. 10.1037/0022-3514.72.1.207 9008382

[B136] Miller N. Pedersen W. C. Earleywine M. Pollock V. E. (2003). A theoretical model of triggered displaced aggression. *Pers. Soc. Psychol. Rev.* 7 75–97. 10.1207/S15327957PSPR0701_5 12584058

[B137] Nash K. Inzlicht M. McGregor I. (2012). Approach-related left prefrontal EEG asymmetry predicts muted error-related negativity. *Biol. Psychol.* 91 96–102. 10.1016/j.biopsycho.2012.05.005 22634389

[B138] Nash K. McGregor I. Inzlicht M. (2010). Line bisection as a neural marker of approach motivation. *Psychophysiology* 47 979–983. 10.1111/j.1469-8986.2010.00999.x 20233339

[B139] Nash K. Prentice M. Hirsh J. McGregor I. Inzlicht M. (2014). Muted neural response to distress among securely attached people. *Soc. Cogn. Affect. Neurosci.* 9 1239–1245. 10.1093/scan/nst099 23887815 PMC4127024

[B140] Nash K. A. McGregor I. Prentice M. (2011). Threat and defense as goal regulation: from implicit goal conflict to anxious uncertainty, reactive approach motivation, and ideological extremism. *J. Pers. Soc. Psychol.* 101 1291–1301. 10.1037/a0025944 22103578

[B141] Newby-Clark I. R. McGregor I. Zanna M. P. (2002). Thinking and caring about cognitive inconsistency: when and for whom does attitudinal ambivalence feel uncomfortable? *J. Pers. Soc. Psychol.* 82 157–166. 10.1037/0022-3514.82.2.157 11831406

[B142] Olvet D. M. Hajcak G. (2008). The error-related negativity (ERN) and psychopathology: toward an endophenotype. *Clin. Psychol. Rev* 28 1343–1354. 10.1016/j.cpr.2008.07.003 18694617 PMC2615243

[B143] Paulhus D. L. (2014). Toward a taxonomy of dark personalities. *Curr. Dir. Psychol. Sci.* 23 421–426. 10.1177/0963721414547737

[B144] Paulhus D. L. Williams K. M. (2002). The dark triad of personality: narcissism, Machiavellianism, and psychopathy. *J. Res. Pers.* 36 556–563. 10.1016/S0092-6566(02)00505-6

[B145] Perreaux L. (2015). *Ten Arrests at Montreal Airport Shine Light on Radicalization Problem. The Globe and Mail.* Available at: http://www.theglobeandmail.com/news/national/rcmp-arrests-10-at-montreal-airport-suspected-of-wanting-to-join-jihadists/article24509383/ [accessed May 21 2015].

[B146] Post J. M. (1990). “Terrorist psycho-logic: terrorist behavior as a product of psychological forces,” in *Origins of Terrorism: Psychologies, Ideologies, Theologies, States of Mind*, ed. Reich W. (New York, NY: Cambridge University Press), 25–40.

[B147] Post J. M. (2005). When hatred is bred in the bone: psycho-cultural foundations of contemporary terrorism. *Polit. Psychol.* 26 615–636. 10.1111/j.1467-9221.2005.00434.x

[B148] Postmes T. Spears R. (1998). Deindividuation and antinormative behavior: a meta-analysis. *Psychol. Bull.* 123 238–259. 10.1037/0033-2909.123.3.238

[B149] Proulx T. Inzlicht M. Harmon-Jones E. (2012). Understanding all inconsistency compensation as a palliative response to violated expectations. *Trends Cogn. Sci. (Regul. Ed.)* 16 285–291. 10.1016/j.tics.2012.04.002 22516239

[B150] Pyszczynski T. Abdollahi A. Solomon S. Greenberg J. Cohen F. Weise D. (2006). Mortality salience, martyrdom, and military might: the great satan versus the axis of evil. *Pers. Soc. Psychol. Bull.* 32 525–537. 10.1177/0146167205282157 16513804

[B151] Qatar International Academy for Security Studies. (2010). *Risk Reduction for Countering Violent Extremism.* Available at: http://soufangroup.com/risk-reduction-for-countering-violentextremism/?catid=23

[B152] Rogers C. R. (1951). *Client-Centered Therapy.* Cambridge: Riverside.

[B153] Rothschild Z. K. Abdollahi A. Pyszczynski T. (2009). Does peace have a prayer? The effect of mortality salience, compassionate values, and religious fundamentalism on hostility toward out-groups. *J. Exp. Soc. Psychol.* 45 816–827. 10.1016/j.jesp.2009.05.016

[B154] Ryan R. M. Deci E. L. (2000). Self-determination theory and the facilitation of intrinsic motivation, social development, and well-being. *Am. Psychol.* 55 68–78. 10.1037/0003-066X.55.1.68 11392867

[B155] Sageman M. (2011). *Leaderless Jihad: Terror Networks in the Twenty-First Century.* Philadelphia, PA: University of Pennsylvania Press.

[B156] Sageman M. (2014). The stagnation in terrorism research. *Terror. Polit. Violence* 26 565–580. 10.1080/09546553.2014.895649

[B157] Sartre J. P. (1956). *Being and Nothingness.* trans. Barnes H. New York, NY: Pocket Books.

[B158] Sassenrath C. Sassenberg K. Scholl A. (2014). From a distance… the impact of approach and avoidance motivational orientation on perspective taking. *Soc. Psychol. Personal. Sci.* 5 18–26. 10.1177/1948550613486672

[B159] Schmeichel B. J. Gailliot M. T. Filardo E.-A. McGregor I. Gitter S. Baumeister R. F. (2009). Terror management theory and self-esteem revisited: the roles of implicit and explicit self-esteem in mortality salience effects. *J. Pers. Soc. Psychol.* 96 1077–1087. 10.1037/a0015091 19379037

[B160] Schumann K. McGregor I. Nash K. Ross M. (2014). Religious magnanimity: reminding people of their religious belief system reduces hostility after threat. *J. Pers. Soc. Psychol.* 107 432–453. 10.1037/a0036739 25133725

[B161] Shah J. Y. Friedman R. Kruglanski A. W. (2002). Forgetting all else: on the antecedents and consequences of goal shielding. *J. Pers. Soc. Psychol.* 83 1261–1280. 10.1037/0022-3514.83.6.1261 12500810

[B162] Shah J. Y. Kruglanski A. W. Thompson E. P. (1998). Membership has its (epistemic) rewards: need for closure effects on in-group bias. *J. Pers. Soc. Psychol.* 75 383–393. 10.1037/0022-3514.75.2.383 9731314

[B163] Shariff A. F. Norenzayan A. (2007). God is watching you priming God concepts increases prosocial behavior in an anonymous economic game. *Psychol. Sci.* 18 803–809. 10.1111/j.1467-9280.2007.01983.x 17760777

[B164] Shariff A. F. Willard A. K. Andersen T. Norenzayan A. (2015). Religious priming a meta-analysis with a focus on prosociality. *Pers. Soc. Psychol. Rev.* 10.1177/1088868314568811 [Epub ahead of print]. 25673322

[B165] Sim S. (2013). *Countering Violent Extremism: Leveraging Terrorist Dropouts to Counter Violent Extremism in Southeast Asia.* New York, NY: Qatar International Academy for Security Studies (QIASS).

[B166] Simon L. Arndt J. Greenberg J. Pyszczynski T. Solomon S. (1998). Terror management and meaning: evidence that the opportunity to defend the worldview in response to mortality salience increases the meaningfulness of life in the mildly depressed. *J. Pers.* 66 359–382. 10.1111/1467-6494.00016 9615422

[B167] Sleegers W. W. Proulx T. van Beest I. (2015). Extremism reduces conflict arousal and increases values affirmation in response to meaning violations. *Biol. Psychol.* 108 126–131. 10.1016/j.biopsycho.2015.03.012 25857674

[B168] Smith H. (1986). *The Religions of Man.* New York, NY: Harper and Row.

[B169] Solomon S. Greenberg J. Pyszczynski T. (1991). A terror management theory of social behavior: the psychological functions of self-esteem and cultural worldviews. *Adv. Exp. Soc. Psychol.* 24 93–159. 10.1016/S0065-2601(08)60328-7

[B170] Son Hing L. S. Bobocel D. R. Zanna M. P. McBride M. V. (2007). Authoritarian dynamics and unethical decision making: high social dominance orientation leaders and high right-wing authoritarianism followers. *J. Pers. Soc. Psychol.* 92 67–81. 10.1037/0022-3514.92.1.67 17201543

[B171] Steele C. M. (1988). “The psychology of self-affirmation: sustaining the integrity of the self,” in *Advances in Experimental Social Psychology* Vol. 21 ed. Berkowitz L. (Orlando, FL: Academic Press), 261–302.

[B172] Stollberg J. Fritsche I. Bäcker A. (2015). Striving for group agency: threat to personal control increases the attractiveness of agentic groups. *Front. Psychol.* 6:649 10.3389/fpsyg.2015.00649 PMC4444748 26074832

[B173] Sullivan D. Landau M. J. Rothschild Z. K. (2010). An existential function of enemyship: evidence that people attribute influence to personal and political enemies to compensate for threats to control. *J. Pers. Soc. Psychol.* 98 434–449. 10.1037/a0017457 20175623

[B174] Swann W. B. Buhrmester M. D. (2015). Identity Fusion. *Curr. Dir. Psychol. Sci.* 24 52–57. 10.1177/0963721414551363

[B175] Tagney J. P. Baumeister R. F. Boone A. L. (2004). High self control predicts good adjustment, less pathology, better grades, and interpersonal success. *J. Pers.* 72 271–324. 10.1111/j.0022-3506.2004.00263.x 15016066

[B176] Taylor S. E. Gollwitzer P. M. (1995). Effects of mindset on positive illusions. *J. Pers. Soc. Psychol.* 69 213–226. 10.1037/0022-3514.69.2.213 7643303

[B177] Tesser A. (2000). On the confluence of self-esteem maintenance mechanisms. *Pers. Soc. Psychol. Rev.* 4 290–299. 10.1207/S15327957PSPR0404_1

[B178] The Economist. (2015). *Jihafrica.* Available at: http://www.economist.com/news/middle-east-and-africa/21657801-biggest-threat-african-peace-and-prosperity-comes-dangerous [accessed July 18 2015].

[B179] The Soufan Group. (2013). *Countering Violent Extremism: The Counter Narrative Study.* Available at: http://soufangroup.com/countering-violent-extremism-the-counter-narrative-study/ [accessed October 29 2013].

[B180] The Soufan Group. (2015). *The International Hotbeds of the Islamic State.* Available at: http://soufangroup.com/tsg-intelbrief-the-international-hotbeds-of-the-islamic-state/ [accessed July 22 2015].

[B181] Twenge J. M. Campbell W. K. (2003). “Isn’t it fun to get the respect that we’re going to deserve?” Narcissism, social rejection, and aggression. *Pers. Soc. Psychol. Bull.* 29 261–272. 10.1177/0146167202239051 15272953

[B182] Vallacher R. R. Wegner D. M. (1989). Level of personal agency: individual variation in action identification. *J. Pers. Soc. Psychol.* 57 660–671. 10.1037/0022-3514.57.4.660 2926623

[B183] Vernon P. A. Villani V. C. Vickers L. C. Harris J. A. (2008). A behavioral genetic investigation of the Dark Triad and the Big 5. *Pers. Individ. Dif.* 44 445–452. 10.1016/j.paid.2007.09.007

[B184] Walton G. M. Cohen G. L. (2007). A question of belonging: race, social fit, and achievement. *J. Pers. Soc. Psychol.* 92 82–96. 10.1037/0022-3514.92.1.82 17201544

[B185] Walton G. M. Cohen G. L. (2011). A brief social-belonging intervention improves academic and health outcomes of minority students. *Science* 331 1447–1451. 10.1126/science.1198364 21415354

[B186] Watkins E. Moberly N. J. Moulds M. L. (2008). Processing mode causally influences emotional reactivity: distinct effects of abstract versus concrete construal on emotional response. *Emotion* 8 364–378. 10.1037/1528-3542.8.3.364 18540752 PMC2672048

[B187] Watson P. J. Grisham S. O. Trotter M. V. Biderman M. D. (1984). Narcissism and empathy: validity evidence for the Narcissistic Personality Inventory. *J. Pers. Assess.* 48 301–305. 10.1207/s15327752jpa4803_12 16367529

[B188] Weaver M. (2015). Her majesty’s jihadists. *The New York Times* Available at: http://www.nytimes.com/2015/04/19/magazine/her-majestys-jihadists.html?_r=0 [accessed April 14 2015].

[B189] Webber D. Schimel J. Martens A. Hayes J. Faucher E. H. (2013). Using a bug-killing paradigm to understand how social validation and invalidation affect the distress of killing. *Pers. Soc. Psychol. Bull.* 39 470–481. 10.1177/0146167213477891 23407746

[B190] Wichman A. L. (2010). Uncertainty and religious reactivity: uncertainty compensation, repair, and inoculation. *Eur. J. Soc. Psychol.* 40 35–42.

[B191] Wicklund R. A. Gollwitzer P. M. (1982). *Symbolic Self Completion.* Hillsdale, NJ: Lawrence Erlbaum.

[B192] Wohl M. J. Branscombe N. R. Reysen S. (2010). Perceiving your group’s future to be in jeopardy: extinction threat induces collective angst and the desire to strengthen the ingroup. *Pers. Soc. Psychol. Bull.* 36 898–910. 10.1177/0146167210372505 20519571

